# Tissue-specific inflammatory responses to systemic LPS in mice with LysM-lineage Hmgb1 deletion

**DOI:** 10.1016/j.bbih.2026.101382

**Published:** 2026-10-06

**Authors:** James A. Johnson, Angar Tsoggerel, Kathryn Fischer, Alexandru Movila, Antentor Hinton, Kimberly Sullivan

**Affiliations:** a Department of Biochemistry, Molecular Biology and Pharmacology, The Stark Neurosciences Research Institute, Indiana University School of Medicine, Indianapolis, IN, USA; b Department of Biomedical Science Indiana University School of Dentistry, Indianapolis, IN, USA; c Indiana Center for Musculoskeletal Health, Indiana University School of Medicine, Indianapolis, IN, USA; d Richard L. Roudebush VA Medical Center, Indianapolis, IN, USA; e Department of Molecular Physiology and Biophysics, Vanderbilt University Medical Center, Nashville, USA; f Department of Environmental Health, Boston University School of Public Health, Boston, MA, USA

## Abstract

**Background:**

Systemic lipopolysaccharide (LPS) exposure induces peripheral inflammation that can engage neuroimmune pathways. High-mobility group box 1 (HMGB1) may contribute to peripheral–central communication, but the role of lysozyme M (LysM)-expressing cells in circulating HMGB1 and compartment-specific inflammatory responses remains unclear.

**Methods:**

Male 7–8-week-old *HMGB1*^*fl/fl*^*LysM-Cre*^*+*^ mice and *HMGB1*^*fl/fl*^*LysM-Cre*^*-*^ littermate controls received LPS (5 mg/kg, i.p.) or saline. Plasma HMGB1, midbrain inflammatory gene expression, cervical lymph node (CLN) transcriptional responses, and choroid plexus CD45 immunoreactivity were assessed at 3 h and 7 days. Spleen, liver, and bone marrow transcriptional responses and Y-maze performance were assessed at 7 days.

**Results:**

LPS increased plasma HMGB1 at 3 h and 7 days without significant genotype differences. Despite comparable circulating HMGB1, LysM-lineage *Hmgb1* deletion was associated with altered expression of selected midbrain inflammatory transcripts at both time points and with distinct acute and temporal CLN transcriptional responses. At 7 days, splenic responses largely overlapped between genotypes, whereas liver and bone marrow gene-level responses were limited. LPS increased choroid plexus CD45 immunoreactivity at both time points without genotype effects. Y-maze outcomes showed no significant genotype, treatment, or interaction effects.

**Conclusions:**

LysM-lineage *Hmgb1* deletion is associated with compartment- and time-dependent inflammatory responses following sublethal LPS-induced endotoxemia. The dissociation between plasma HMGB1 and genotype-associated midbrain and CLN responses indicates that total circulating HMGB1 did not fully reflect compartment-specific responses in this model, highlighting the importance of anatomical context in peripheral–central immune signaling.

## Highlights

• LPS elevates plasma HMGB1 at 3 h and 7 days without a significant genotype effect.
• LysM-lineage Hmgb1 deletion alters selected midbrain transcripts at 3 h and 7 d.
• LysM-lineage Hmgb1 deletion modifies acute CLN transcriptional responses.
• Peripheral-organ responses show limited genotype dependence at 7 days.
• LPS increases choroid plexus CD45 immunoreactivity without a significant genotype effect.

## 1 Introduction

Systemic inflammation can influence the central nervous system (CNS) even when the initiating stimulus originates outside the brain (Dantzer et al., 2008; Jin et al., 2024; Qin et al., 2007). Systemic administration of lipopolysaccharide (LPS), a component of the outer membrane of Gram-negative bacteria and a pathogen-associated molecular pattern, is commonly used to investigate this peripheral-to-central communication. Through activation of the Toll-like receptor 4 receptor complex, LPS induces a rapid peripheral cytokine response and acute sickness behavior accompanied by inflammatory signaling within the CNS (Hoshino et al., 1999; Lu et al., 2008; Park et al., 2009; Poltorak et al., 1998; Konsman et al., 2002; Lasselin et al., 2020; Spulber et al., 2012). At higher doses, systemic LPS produces experimental endotoxemia and reproduces selected features of sepsis-associated systemic inflammation, although it does not capture the full complexity of infection-driven clinical sepsis (Fernandez-Martin et al., 2022; Fink, 2014; Lewis et al., 2016; Seemann et al., 2017). Although the acute response to LPS is well characterized, previous work using the same 5 mg/kg intraperitoneal LPS paradigm demonstrated that circulating HMGB1 and selected CNS inflammatory changes remained detectable at 7 days, after the acute peripheral inflammatory response had largely resolved (Garza-Lombo et al., 2021). Building on this 3-h and 7-day LPS paradigm, the present study examined whether conditional Hmgb1 deletion in LysM-expressing cells alters circulating HMGB1 and compartment-specific inflammatory responses.

Because the entry of circulating LPS across an intact blood–brain barrier is limited, its effects on the CNS are mediated largely through indirect neural, vascular, and barrier-associated pathways (Jin et al., 2024; Banks and Robinson, 2010; Kodali et al., 2021; Quan, 2008). Circulating inflammatory mediators can activate cerebral endothelial and brain barrier-associated cells, while vagal sensory pathways and circumventricular organs provide additional routes through which peripheral immune activity can be communicated to the brain. Within this network, the choroid plexus serves as an important neuroimmune interface through its roles in maintaining the blood–cerebrospinal fluid barrier, immune surveillance, cytokine signaling, and leukocyte interactions (Shimada and Hasegawa-Ishii, 2021; Lun et al., 2015). CNS-associated and meningeal lymphatic vessels also transport fluid and immune signals toward the cervical lymph nodes, linking CNS-associated compartments with peripheral immune structures (Ahn et al., 2019; Aspelund et al., 2015; Louveau et al., 2015). These interfaces operate alongside systemic organs such as the spleen, liver, and bone marrow, which coordinate broader immune and hematopoietic responses to systemic inflammation (Fernandez-Martin et al., 2022; Lewis et al., 2016; Seemann et al., 2017). Examining these compartments together can therefore provide insight into how peripheral inflammatory signals are distributed across the peripheral–central immune network.

One mediator that may contribute to this communication is high-mobility group box 1 (HMGB1). HMGB1 is a highly conserved nuclear DNA-binding protein involved in chromatin organization and transcriptional regulation. When released extracellularly by activated, stressed, or damaged cells, HMGB1 functions as a damage-associated molecular pattern that can amplify inflammatory signaling through receptors including Toll-like receptors and the receptor for advanced glycation end products (Andersson and Tracey, 2011; Tang et al., 2011; Venereau et al., 2015). Early extracellular HMGB1 may reflect the release of pre-existing intracellular protein, whereas later elevations can result from active secretion by stimulated immune cells and passive release from injured or dying cells (Bonaldi et al., 2003; Gardella et al., 2002; Scaffidi et al., 2002; Wang et al., 1999). These distinct release mechanisms position HMGB1 as a potential link between the initial systemic response to LPS and inflammatory signaling detected at later time points.

The cellular populations contributing to circulating HMGB1 during systemic inflammation remain incompletely defined. Monocytes, macrophages, and granulocytes can release HMGB1 following inflammatory activation, but immune and nonimmune populations may collectively contribute to the circulating pool (Andersson and Tracey, 2011; Tang et al., 2011; Bonaldi et al., 2003; Gardella et al., 2002; Scaffidi et al., 2002; Wang et al., 1999; Vogel et al., 2015). Moreover, the effects of HMGB1 may depend on the tissue in which it is released or acts, making circulating concentrations alone insufficient to describe its compartment-specific functions. Determining how HMGB1 associated with a defined immune-cell lineage influences both circulating HMGB1 and tissue responses may therefore help clarify its role in peripheral-to-central inflammatory communication.

This conditional *HMGB1*^*fl/fl*^ *LysM-Cre* ^*+*^ model was previously used by our laboratory to investigate how HMGB1 associated with peripheral LysM-expressing cells contributes to peripheral–central inflammatory signaling (Greve et al., 2023). Building on that work and the established 3-h and 7-day LPS paradigm (Garza-Lombo et al., 2021), the present study examined how LysM-lineage Hmgb1 deletion influences acute and later responses to a single systemic LPS challenge (Fig. 1). *HMGB1*^*fl/fl*^ *LysM-Cre* ^*+*^ mice and *HMGB1*^*fl/fl*^ *LysM-Cre*^*-*^ littermate controls were used to determine whether this deletion measurably alters total circulating HMGB1, selected inflammatory transcripts in the midbrain, transcriptional responses in the cervical lymph nodes and peripheral immune organs, or CD45 immunoreactivity in the choroid plexus. Spontaneous alternation and locomotor activity were also assessed at 7 days. By comparing central, lymphatic, barrier-associated, and systemic compartments at acute and later time points, the study sought to determine whether the effects of LysM-lineage Hmgb1 deletion depend on tissue context and time following systemic immune activation.

**Fig. 1:**
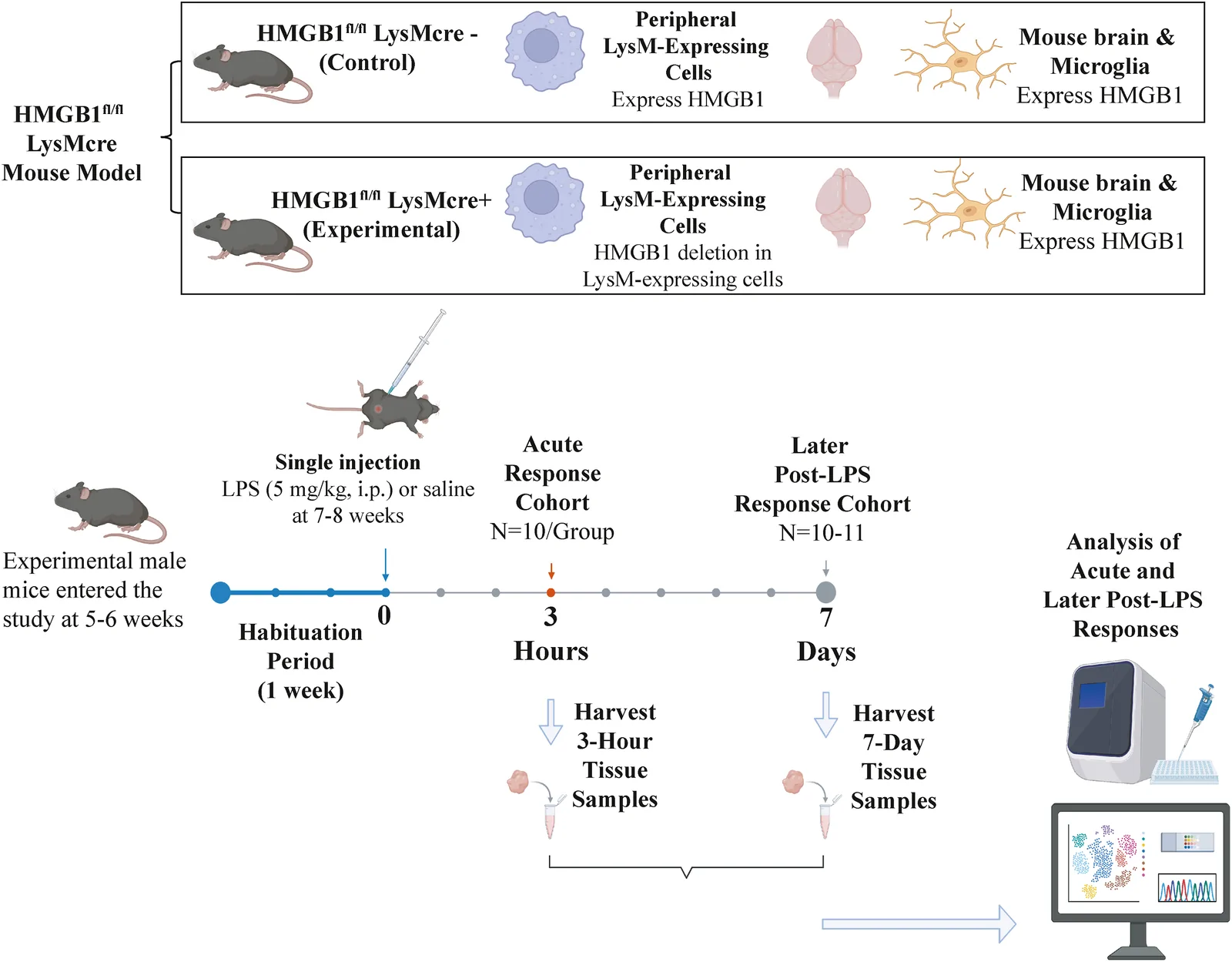
**Experimental design for assessing compartment-specific inflammatory responses following systemic LPS exposure in mice with LysM-lineage *Hmgb1* deletion.** Male *HMGB1*^*fl/fl*^*LysM-cre*^*+*^ mice with conditional *Hmgb1* deletion in LysM-expressing cells and *HMGB1*^*fl/fl*^*LysM-cre*^*-*^ littermate controls received a single intraperitoneal injection of LPS (5 mg/kg) or saline. Samples were collected at 3 h to assess acute inflammatory responses and at 7 days to assess later post-exposure responses. Plasma, brain, cervical lymph nodes, spleen, liver, bone marrow, and choroid plexus were evaluated using the corresponding biochemical, molecular, transcriptomic, and immunofluorescence analyses described in the Methods.

## 2 Materials and methods

### 2.1 Reagents

Lipopolysaccharide (LPS; strain O111:B4) was obtained from EMD Chemicals (Gibbstown, NJ, USA) and used to induce systemic inflammation. Cytokine levels were quantified using commercial ELISA kits. IL-1β ELISA kits were obtained from R&D Systems (Minneapolis, MN, USA), and HMGB1 ELISA kits were obtained from CUSABIO (Wuhan, China). We performed all assays according to the manufacturers’ instructions. We used the nCounter Mouse Immunology and Mouse Inflammation Panels (Bruker Spatial Biology, formerly NanoString Technologies, Billerica, MA, USA) for transcriptomic analyses. Antibodies used for immunofluorescence, including manufacturers catalog information, are listed in Supplementary Table S1A.

### 2.2 Mice

Homozygous HMGB1 floxed mice (B6.129P2-Hmgb1ˆtm1Ttgˆ; RIKEN BioResource Research Center, RBRC06240) were crossed with LysM-Cre mice (B6.129P2-Lyz2ˆtm1(cre)Ifo/J; The Jackson Laboratory, stock no. 004781) to generate *HMGB1*^*fl/fl*^ *LysM-Cre* ^+/+^ mice with conditional Hmgb1 deletion in LysM-expressing cells, as previously described (Greve et al., 2023; Ahmed et al., 2024). *HMGB1*^*fl/fl*^ *LysM-Cre*^−/−^ littermates lacking the LysM-Cre allele served as genotype-matched controls. The colony was maintained on a C57BL/6 genetic background. LysM-Cre-mediated recombination occurs during development in LysM-expressing cells (Clausen et al., 1999). **For brevity**, ***HMGB1*^fl/fl^*LysM-Cre*^*+/+*^*and HMGB1*^fl/fl^ LysM-Cre^−/−^ mice are hereafter referred to as LysM-Cre^+^ and LysM-Cre^-^ mice, respectively.**

Genotypes were verified by PCR analysis of genomic DNA isolated from ear notch tissue using the Qiagen DNeasy Blood & Tissue Kit. Separate PCR assays were used to identify the floxed Hmgb1 allele and the LysM-Cre allele. Primer sequences, genotyping reagents, and genotype-identification criteria are provided in Supplementary Tables S1E, S1F, and S1G, respectively.

Male mice entered the study and began habituation at 5–6 weeks of age and were 7–8 weeks old at the time of LPS or saline administration. Animals were housed individually in an AAALAC-accredited facility under a 12-h light/dark cycle at an ambient temperature of 20–24 °C, with lights on at 07:00 h and lights off at 19:00 h. Food and water were available ad libitum.

All procedures were approved by the Indiana University School of Medicine Institutional Animal Care and Use Committee (protocol numbers #24046 and #20092) and were conducted in accordance with National Institutes of Health guidelines for the care and use of laboratory animals. Two independent experiments were performed. In 3-h and 7-day experiments, *HMGB1*^*fl/fl*^ LysM-Cre^+/+^ mice and *HMGB1*^f*l/fl*^ LysM-Cre^−/−^ littermate controls were randomly assigned to saline or LPS treatment, with 10 mice per genotype × treatment group and 10–11 mice per genotype × treatment group at 7 days.

### 2.3 Systemic LPS exposure and tissue collection

Lipopolysaccharide (LPS; strain O111:B4) was prepared in sterile 0.9% saline immediately before use. Mice received a single intraperitoneal injection of LPS (5 mg/kg) or an equivalent volume of sterile 0.9% saline to induce systemic inflammation and experimental endotoxemia (Qin et al., 2007; Fernandez-Martin et al., 2022). This high-dose LPS challenge reproduces selected features of sepsis-associated systemic inflammation but does not recapitulate the full complexity of infection-driven clinical sepsis. Animals were monitored after injection for signs of sickness behavior. Mice were euthanized at 3 h to assess the acute response or at 7 days to assess later post-exposure responses.

The right hemisphere was immersion-fixed in 4% paraformaldehyde (PFA) for histological analyses and subsequently cryoprotected in 30% sucrose. From the left hemisphere, the midbrain, cortex, hippocampus, and cerebellum were carefully dissected and snap-frozen for molecular analyses.

Bone marrow was collected by flushing the femurs and tibias with sterile phosphate-buffered saline (PBS). Bone marrow mononuclear cells were then isolated using Histopaque density-gradient centrifugation (Sigma-Aldrich, St. Louis, MO, USA). Total RNA was extracted from the isolated cells and stored at −80 °C until NanoString gene-expression analysis. Cervical lymph nodes (CLNs), liver, spleen, and blood were also collected for downstream analyses, and samples were stored at −80 °C until use.

### 2.4 Behavioral testing (Y-maze spontaneous alternation task)

Working memory and exploratory activity were assessed 7 days after saline or LPS administration using a Y-maze spontaneous alternation task. The maze consisted of three identical opaque white arms (35 × 20 × 8 cm) positioned at 120° angles. Behavioral testing was conducted under dim illumination (∼4 lux) with continuous white noise to minimize external disturbances.

Male *HMGB1*^*fl/fl*^ *LysM-Cre*^*-*^ and *HMGB1*^*fl/fl*^ *LysM-Cre* ^*+*^ mice received either saline or LPS (n = 9–11 per group). Before testing, mice were allowed to acclimate to the behavioral testing room for 30 min. Each mouse was placed at the end of one arm and allowed to freely explore the maze for 10 min. An arm entry was defined as entry with all four paws into an arm. The sequence of arm entries was recorded, and spontaneous alternation was calculated as the percentage of consecutive entries into three different arms using the formula: (number of alternations/[total arm entries − 2]) × 100. Total distance traveled was used as an index of locomotor activity.

The maze was cleaned with 70% ethanol between trials to minimize olfactory cues. Behavioral testing was performed between 09:00 and 13:00 h in a quiet, temperature-controlled room maintained at 20–24 °C. The animal facility was maintained on a 12-h light/dark cycle, with lights on at 07:00 h and lights off at 19:00 h; therefore, behavioral testing occurred during the light phase. A blinded experimenter conducted all behavioral assessments. Behavioral data were analyzed using two-way analysis of variance (ANOVA) with genotype and treatment as factors in GraphPad Prism version 9.5.0 (GraphPad Software, San Diego, CA, USA).

### 2.5 Enzyme-linked immunosorbent assay (ELISA)

Blood samples were collected into EDTA-coated tubes and centrifuged at 1500 × g for 10 min at 4 °C to isolate plasma. Plasma samples were aliquoted and stored at −80 °C until analysis. Concentrations of IL-1β and HMGB1 in plasma were measured using commercially available ELISA kits according to the manufacturers’ instructions (see Supplementary Table S1D for reagent details). Briefly, 100 μL of standards or plasma samples were added to each well and incubated for 2 h at 37 °C. Plates were washed, and 100 μL of biotinylated detection antibody was added and incubated for 1 h at 37 °C. After washing, 100 μL of HRP-conjugated avidin was added and incubated for an additional 1 h at 37 °C. The reaction was developed using TMB substrate, and absorbance was measured at 450 nm using a SpectraMax i3 plate reader (Molecular Devices, San Jose, CA, USA).

### 2.6 Quantitative reverse-transcription PCR (qRT-PCR)

Total RNA was extracted from the midbrain using TRIzol™ reagent (Thermo Fisher Scientific, Waltham, MA, USA) and treated with DNase I to remove genomic DNA contamination. Complementary DNA (cDNA) was synthesized using a first-strand cDNA synthesis kit. Quantitative PCR (see reagent list in Supplementary Table S1D) was performed using PowerUp SYBR Green chemistry or TaqMan Fast Advance Master Mix on a Viia7 RT-PCR system. Gene-specific primers are listed in Supplementary Table S1B or TaqMan probes Supplementary Table S1C. Relative gene expression levels were normalized to *Gapdh* and calculated using the 2^−ΔΔCt^ method.

### 2.7 Immunohistochemistry and immunofluorescence

Right brain hemispheres were immersion-fixed in 4% paraformaldehyde for 24 h at 4 °C and subsequently cryoprotected in 30% sucrose at 4 °C for 24–48 h, or until the tissue sank. Fixed brains were sectioned sagittally at 40 μm using a freezing microtome to obtain free-floating sections containing the lateral ventricle and choroid plexus. Sections were stored in PBS containing 0.02% sodium azide at 4 °C until immunostaining.

Before staining, sections were permeabilized in 0.1% Triton X-100 and subjected to heat-mediated antigen retrieval in sodium citrate buffer (pH 6.0) at 95 °C for 10 min. Sections were then blocked with 5% normal goat serum and incubated overnight at 4 °C with primary antibodies against CD45 and cytokeratin (see Supplementary Table S1A for antibody details).

After washing, sections were incubated with species-appropriate Alexa Fluor–conjugated secondary antibodies, and nuclei were counterstained with DAPI. Negative-control sections were processed without primary antibodies but were incubated with the corresponding secondary antibodies and DAPI under otherwise identical conditions. Following staining, sections were mounted on glass slides and coverslipped using antifade mounting medium.

Images were acquired using a Leica DM6 B Thunder fluorescence microscope (Leica Microsystems, Wetzlar, Germany) with a 20× objective. Z-stacks were collected at 1-μm intervals across the choroid plexus. CD45 immunoreactivity was quantified by a blinded investigator using Fiji-ImageJ software. Mean CD45 fluorescence intensity was measured within manually defined choroid plexus regions of interest following background subtraction.

### 2.8 NanoString nCounter gene expression analysis

NanoString Mouse Immunology Panels were used to analyze cervical lymph-node (CLN) samples collected at 3 h and 7 days and bone-marrow samples collected at 7 days. Mouse Inflammation Panels were used to analyze liver and spleen samples collected at 7 days. For each tissue and time point, three independent biological samples were analyzed per genotype × treatment group (n = 3/group). Each 12-sample cartridge included the four genotype × treatment groups, with three biological samples per group.

Total RNA (75 ng/sample) was hybridized to the corresponding probe sets according to the manufacturer's protocol and processed using the nCounter MAX/FLEX system (Bruker Spatial Biology, formerly NanoString Technologies, Billerica, MA, USA). Raw counts were imported into nSolver Analysis Software version 4.0.70 with the nCounter Advanced Analysis module (Bruker Spatial Biology) for data processing. Background correction and positive-control normalization were performed according to the manufacturer's guidelines.

For the Mouse Immunology Panel, transcript counts were normalized to the geometric mean of the housekeeping genes *G6pdx*, *Tbp*, *Sdha*, *Eef1g*, *Rpl19*, *Gapdh*, *Oaz1*, *Ppia*, and *Polr2a*. For the Mouse Inflammation Panel, normalization was performed using *Tubb5*, *Gapdh*, *Cltc*, *Gusb*, *Pgk1*, and *Hprt*. Housekeeping genes were selected according to their expression stability using the geNorm algorithm implemented in the nCounter Advanced Analysis module.

Differential gene-expression analysis was performed using linear regression models in the nCounter Advanced Analysis module. Genotype, treatment, or both were included as predictor variables, as appropriate for each comparison, and no additional covariates were included. P values were adjusted for multiple comparisons using the Benjamini–Hochberg (BH) method. Genes with a BH-adjusted p value < 0.05 were considered significantly differentially expressed. Log_2_ fold change was used to indicate the direction and magnitude of gene-expression differences and was not applied as an additional significance threshold.

Volcano plots display log_2_ fold change on the x-axis and −log_10_(BH-adjusted p value) on the y-axis. The horizontal reference line at 1.301 corresponds to a BH-adjusted p value of 0.05; genes above this line met the adjusted statistical-significance criterion. Venn diagrams were used to distinguish significantly regulated genes shared between comparisons from those unique to an individual comparison.

Gene-set and pathway analyses were performed using the Gene Set Analysis and pathway-scoring tools implemented in the nCounter Advanced Analysis module. Additional data visualization was performed using GraphPad Prism version 9.5.0 (GraphPad Software, San Diego, CA, USA).

### 2.9 Statistical analysis

Sample sizes were determined based on prior studies and power analyses (Seemann et al., 2017; Deng et al., 2019; Yamada et al., 2026; Matsuura et al., 2023). A randomized block design was used, and sample processing and analysis were performed by investigators blinded to treatment group. Data are presented as mean ± SEM. Behavioral outcomes from the Y-maze and plasma cytokine concentrations measured by ELISA were analyzed using two-way analysis of variance (ANOVA) with genotype and treatment as factors, followed by Bonferroni post hoc tests when appropriate. Comparisons between two groups were analyzed using two-tailed Student's *t* tests. NanoString differential expression analyses were performed using linear regression models within the nSolver Advanced Analysis module, with genotype and/or treatment included as predictor variables depending on the comparison. P-values were adjusted for multiple testing using the Benjamini–Hochberg (BH) method, and genes with an adjusted *P* value < 0.05 were considered statistically significant.

All statistical analyses were conducted using GraphPad Prism version 9.5.0 (GraphPad Software, San Diego, CA, USA), unless otherwise specified. Complete statistical results, including F statistics, degrees of freedom, exact p-values, and post hoc multiple comparisons, are provided in the corresponding Supplementary Tables. Exact sample sizes for each analysis are indicated in the corresponding figure legends and Supplementary Tables.

## 3 Results

### 3.1 Y-maze performance in mice with LysM-lineage Hmgb1 deletion 7 days after LPS exposure

To determine whether LysM-lineage *Hmgb1* deletion influences exploratory activity or spontaneous alternation during the later phase following systemic inflammation, mice were assessed in the Y-maze 7 days after LPS administration. This time point represented the later post-LPS assessment used to evaluate behavioral and inflammatory outcomes in the study.

The Y-maze assesses exploratory behavior and spontaneous alternation, which is commonly used as an index of spatial working memory (Kraeuter et al., 2019). Two-way ANOVA revealed no significant main effects of genotype (F(1,35) = 0.014, P = 0.907) or treatment (F(1,35) = 1.985, P = 0.168), and no genotype × treatment interaction (F(1,35) = 0.027, P = 0.871) for spontaneous alternation percentage. Similarly, analysis of total distance traveled revealed no significant effects of genotype (F(1,35) = 0.012, P = 0.913), treatment (F(1,35) = 0.725, P = 0.400), or genotype × treatment interaction (F(1,35) = 1.289, P = 0.264). Total arm entries were also comparable across groups. Behavioral data are presented in Supplementary Fig. S1.

### 3.2 Circulating HMGB1 levels following LPS exposure in mice with LysM-lineage *Hmgb1* deletion

We next examined whether LysM-lineage Hmgb1 deletion was associated with changes in circulating HMGB1 levels following systemic LPS exposure. To assess systemic HMGB1 responses, plasma HMGB1 levels were measured by ELISA. At 3 h, two-way ANOVA revealed a significant main effect of treatment on plasma HMGB1 levels (F(1,32) = 64.68, P < 0.0001), with no significant main effect of genotype (F(1,32) = 0.4107, P = 0.5262) or genotype × treatment interaction (F(1,32) = 0.6693, P = 0.4194). Bonferroni post hoc comparisons showed significantly higher plasma HMGB1 levels following LPS compared with saline in both *HMGB1*^*fl/fl*^ *LysM-Cre*-control mice and *HMGB1*^*fl/fl*^ *LysM-Cre* ^+^ mice (adjusted P < 0.0001 for both comparisons; Fig. 2A). No significant difference was observed between genotypes within the LPS-treated groups (adjusted P > 0.9999).

**Fig. 2:**
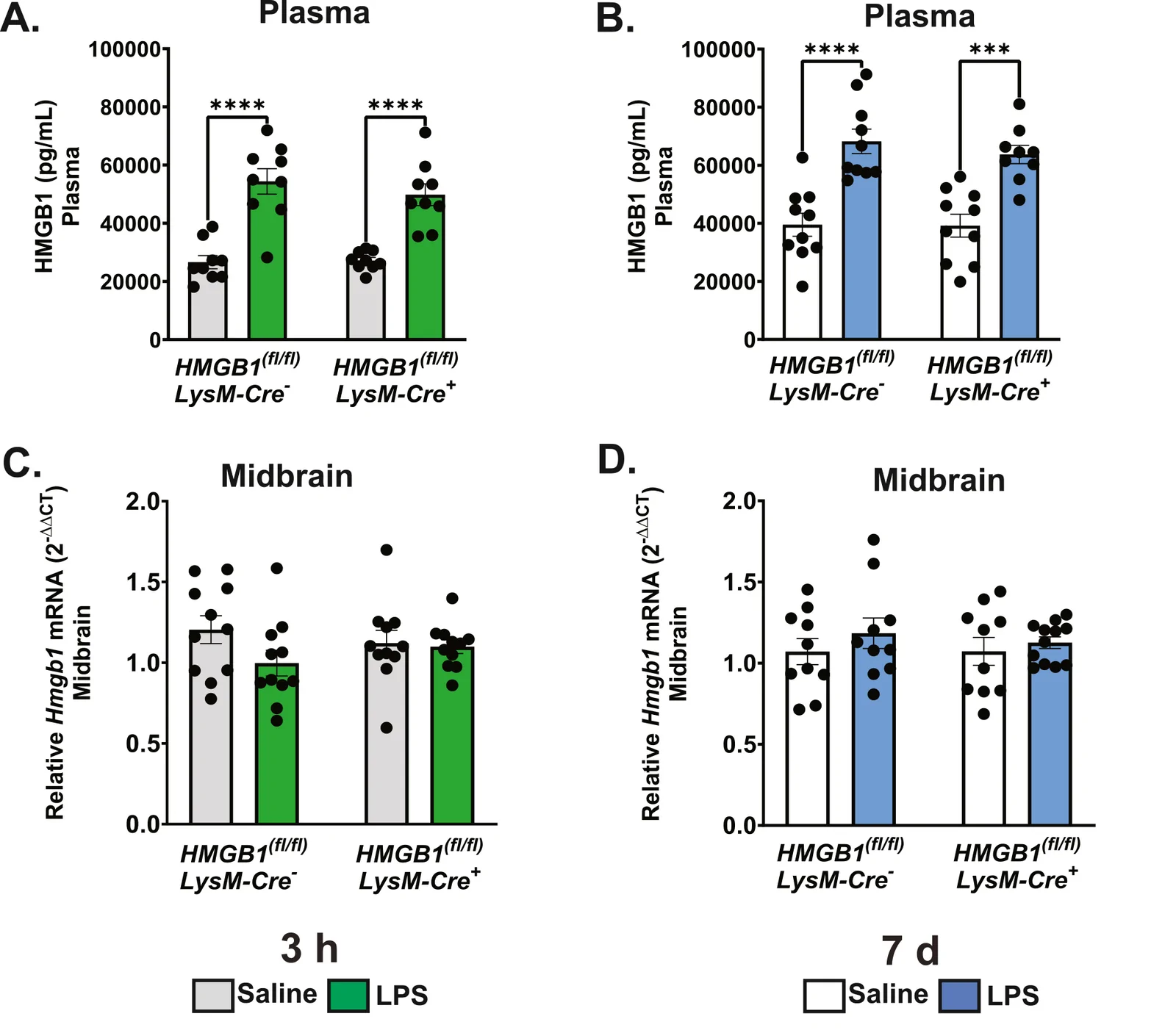
**Circulating HMGB1 and midbrain *Hmgb1* expression following systemic LPS exposure in mice with LysM-lineage *Hmgb1* deletion.** Male *HMGB1*^*fl/fl*^*LysM-cre*^*+*^ mice with conditional *Hmgb1* deletion in LysM-expressing cells and *HMGB1*^*fl/fl*^*LysM-cre*^*-*^ littermate controls received a single intraperitoneal injection of LPS (5 mg/kg) or saline. Plasma and midbrain tissues were collected at 3 h and 7 days post-injection. Plasma HMGB1 concentrations were quantified by ELISA at 3 h (**A**) and 7 days (**B**). Midbrain *Hmgb1* mRNA expression was measured by qRT-PCR at 3 h (**C**) and 7 days (**D**), normalized to *Gapdh*, and calculated using the 2^−ΔΔCT^ method. Data are presented as mean ± SEM (n = 9–11 mice per group). Statistical analyses were performed using two-way ANOVA with genotype and treatment as factors, followed by Bonferroni post hoc comparisons. Complete statistical results are provided in Supplementary Table S3A–S3B. ***P < 0.001; ****P < 0.0001, LPS versus saline.

At 7 days, plasma HMGB1 levels also showed a significant main effect of treatment (F(1,35) = 47.11, P < 0.0001), with no significant main effect of genotype (F(1,35) = 0.3928, P = 0.5349) or genotype × treatment interaction (F(1,35) = 0.2952, P = 0.5903). Bonferroni post hoc comparisons showed significantly higher plasma HMGB1 levels following LPS compared with saline in *HMGB1*^*fl/fl*^ *LysM-Cre*^-^ control mice (adjusted P < 0.0001) and *HMGB1*^*fl/fl*^ *LysM-Cre* ^+^ mice (adjusted P = 0.0006; Fig. 2B). No significant difference was observed between genotypes within the LPS-treated groups (adjusted P > 0.9999).

Midbrain Hmgb1 mRNA expression was not significantly altered at either time point. At 3 h, there were no significant main effects of treatment (F(1,40) = 2.404, P = 0.1289) or genotype (F(1,40) = 0.01534, P = 0.9020), and no significant genotype × treatment interaction (F(1,40) = 1.570, P = 0.2174; Fig. 2C). Similarly, at 7 days, there were no significant main effects of treatment (F(1,38) = 1.249, P = 0.2708) or genotype (F(1,38) = 0.1429, P = 0.7075), and no significant genotype × treatment interaction (F(1,38) = 0.1591, P = 0.6923; Fig. 2D).

Together, these findings indicate that LysM-lineage Hmgb1 deletion did not significantly alter total plasma HMGB1 levels following systemic LPS exposure at the time points examined. We therefore next examined whether LysM-lineage Hmgb1 deletion was associated with changes in inflammatory responses in the midbrain.

### 3.3 LysM-lineage *Hmgb1* deletion is associated with altered peripheral and central cytokine-related responses following LPS exposure

To examine whether LysM-lineage Hmgb1 deletion was associated with altered peripheral and central inflammatory responses following systemic LPS exposure, we measured plasma IL-1β concentrations by ELISA and cytokine-related gene expression in the midbrain by qRT-PCR in *HMGB1*^*fl/fl*^ *LysM-Cre*-control mice and *HMGB1*^*fl/fl*^ *LysM-Cre*+mice (Fig. 3).

**Fig. 3:**
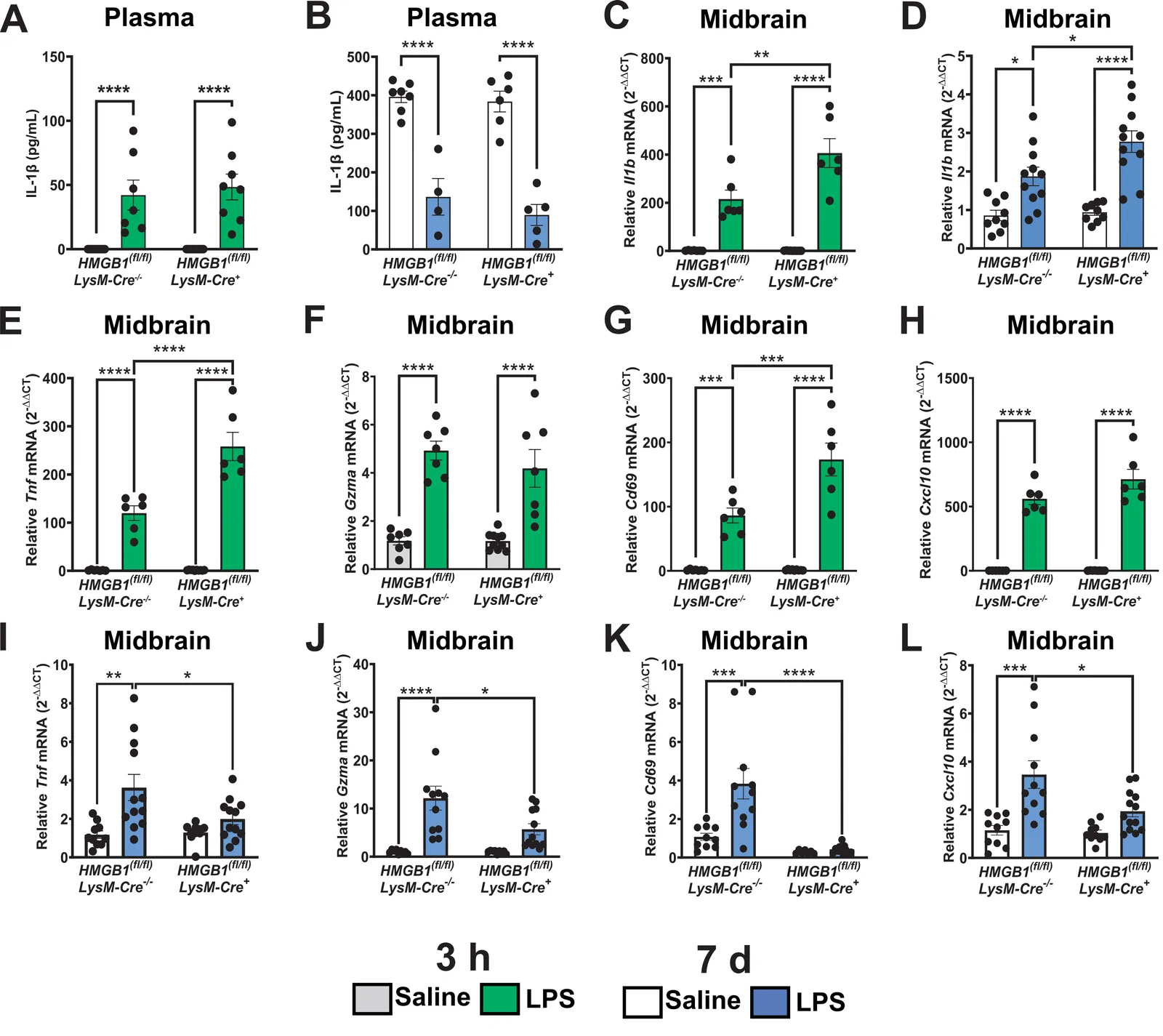
**Cytokine-related responses following systemic LPS exposure in mice with LysM-lineage *Hmgb1* deletion.** Male *HMGB1*^*fl/fl*^*LysM-cre*^*+*^ and *HMGB1*^*fl/fl*^*LysM-cre*^*-*^ mice received a single intraperitoneal injection of LPS (5 mg/kg) or saline. Samples were collected at 3 h (A, C, E–H) and 7 days (B, D, I–L) after LPS or saline administration. Plasma IL-1β concentrations were measured by ELISA at 3 h (A) and 7 days (B). Midbrain *Il1b* expression was measured by qRT-PCR at 3 h (C) and 7 days (D). Additional cytokine-related transcripts measured in the midbrain included *Tnf* (E, I), *Gzma* (F, J), *Cd69* (G, K), and *Cxcl10* (H, L). qRT-PCR expression was normalized to *Gapdh* and is presented as relative expression calculated using the 2^−ΔΔCt^ method; statistical analyses of qRT-PCR data were performed using ΔCt values. Data are presented as mean ± SEM. Sample sizes varied by analysis; exact sample sizes are provided in Supplementary Table S4A–S4G. Statistical analysis was performed using two-way ANOVA with genotype and treatment as factors, followed by Bonferroni post hoc multiple-comparison tests when appropriate. Brackets indicate Bonferroni-adjusted post hoc comparisons: *P < 0.05, **P < 0.01, ***P < 0.001, and ****P < 0.0001.

At 3 h post-LPS, plasma IL-1β concentrations showed a significant main effect of treatment (F(1,31) = 47.48, P < 0.0001), with no significant main effect of genotype (F(1,31) = 0.2315, P = 0.6338) or genotype × treatment interaction (F(1,31) = 0.2315, P = 0.6338). Bonferroni post hoc comparisons showed significantly higher plasma IL-1β concentrations following LPS compared with saline in both *HMGB1*^*fl/fl*^ *LysM-Cre*-(adjusted P = 0.0006) and *HMGB1*^*fl/fl*^ *LysM-Cre*+mice (adjusted P < 0.0001), with no significant difference between genotypes following LPS (adjusted P > 0.9999).

In the midbrain, *Il1b* expression showed significant main effects of treatment (F(1,24) = 104.8, P < 0.0001) and genotype (F(1,24) = 9.907, P = 0.0044), as well as a significant genotype × treatment interaction (F(1,24) = 10.00, P = 0.0042). Bonferroni post hoc comparisons showed significantly higher *Il1b* expression following LPS compared with saline in both *HMGB1*^*fl/fl*^ *LysM-Cre*^-^ (adjusted P = 0.0003) and *HMGB1*^*fl/fl*^ *LysM-Cre*+mice (adjusted P < 0.0001), with significantly lower expression in LysM-Cre^+^ compared with LysM-Cre^-^ mice following LPS (adjusted P = 0.0020). *Tnf* expression similarly showed significant main effects of treatment (F(1,25) = 185.7, P < 0.0001) and genotype (F(1,25) = 25.29, P < 0.0001), as well as a significant genotype × treatment interaction (F(1,25) = 25.20, P < 0.0001). Bonferroni post hoc comparisons showed significantly higher *Tnf* expression following LPS in both genotypes (adjusted P < 0.0001 for both), with significantly lower expression in *HMGB1*^*fl/fl*^ *LysM-Cre*+compared with *HMGB1*^*fl/fl*^ *LysM-Cre*-mice following LPS (adjusted P < 0.0001).

*Cxcl10* expression showed a significant main effect of treatment (F(1,22) = 240.8, P < 0.0001), but no significant main effect of genotype (F(1,22) = 3.510, P = 0.0744) or genotype × treatment interaction (F(1,22) = 3.464, P = 0.0761). *Cd69* expression showed significant main effects of treatment (F(1,24) = 114.7, P < 0.0001) and genotype (F(1,24) = 13.20, P = 0.0013), as well as a significant genotype × treatment interaction (F(1,24) = 13.14, P = 0.0013). Bonferroni post hoc comparisons showed significantly lower *Cd69* expression in *HMGB1*^*fl/fl*^ *LysM-Cre*+compared with *HMGB1*^*fl/fl*^ *LysM-Cre*-mice following LPS (adjusted P = 0.0004). *Gzma* expression showed a significant main effect of treatment (F(1,27) = 67.78, P < 0.0001), but no significant main effect of genotype (F(1,27) = 0.8284, P = 0.3708) or genotype × treatment interaction (F(1,27) = 0.7872, P = 0.3828).

At 7 days post-LPS, plasma IL-1β concentrations showed a significant main effect of treatment (F(1,18) = 99.48, P < 0.0001), with no significant main effect of genotype (F(1,18) = 1.132, P = 0.3013) or genotype × treatment interaction (F(1,18) = 0.3928, P = 0.5387). Bonferroni post hoc comparisons showed significantly lower plasma IL-1β concentrations following LPS compared with saline in both *HMGB1*^*fl/fl*^ *LysM-Cre*^-^ and *HMGB1*^*fl/fl*^ *LysM-Cre*+mice (adjusted P < 0.0001 for both), with no significant difference between genotypes following LPS (adjusted P > 0.9999).

Midbrain *Il1b* expression showed significant main effects of treatment (F(1,36) = 42.87, P < 0.0001) and genotype (F(1,36) = 5.155, P = 0.0293), but no significant genotype × treatment interaction (F(1,36) = 3.553, P = 0.0675). Bonferroni post hoc comparisons showed significantly higher *Il1b* expression following LPS in both *HMGB1*^*fl/fl*^ *LysM-Cre*-(adjusted P = 0.0132) and *HMGB1*^*fl/fl*^ *LysM-Cre* ^+^ mice (adjusted P < 0.0001), with significantly lower expression in *HMGB1*^*fl/fl*^ *LysM-Cre*+compared with *HMGB1*^*fl/fl*^ *LysM-Cre*-mice following LPS (adjusted P = 0.0226).

*Tnf* expression showed a significant main effect of treatment (F(1,40) = 13.87, P = 0.0006) and a significant genotype × treatment interaction (F(1,40) = 4.215, P = 0.0466), but no significant main effect of genotype (F(1,40) = 3.225, P = 0.0801). *Cxcl10* expression showed significant main effects of treatment (F(1,40) = 22.73, P < 0.0001) and genotype (F(1,40) = 5.910, P = 0.0196), as well as a significant genotype × treatment interaction (F(1,40) = 4.403, P = 0.0422). *Cd69* expression showed significant main effects of treatment (F(1,36) = 10.10, P = 0.0030) and genotype (F(1,36) = 21.17, P < 0.0001), as well as a significant genotype × treatment interaction (F(1,36) = 8.131, P = 0.0072). *Gzma* expression also showed significant main effects of treatment (F(1,37) = 27.40, P < 0.0001) and genotype (F(1,37) = 4.573, P = 0.0391), as well as a significant genotype × treatment interaction (F(1,37) = 4.528, P = 0.0401).

Bonferroni post hoc comparisons at 7 days showed significantly higher expression following LPS compared with saline in LysM-Cre^-^ mice for *Tnf* (adjusted P = 0.0012), *Cxcl10* (adjusted P = 0.0001), *Cd69* (adjusted P = 0.0005), and *Gzma* (adjusted P < 0.0001), whereas the corresponding LPS-versus-saline comparisons were not significant in *HMGB1*^*fl/fl*^ *LysM-Cre*+mice (*Tnf,* adjusted P > 0.9999; *Cxcl10*, adjusted P = 0.3689; Cd69, adjusted P > 0.9999; *Gzma*, adjusted P = 0.1966). Following LPS, expression was significantly lower in *HMGB1*^*fl/fl*^ *LysM-Cre* ^+^ compared with *HMGB1*^*fl/fl*^ *LysM-Cre*-mice for *Tnf* (adjusted P = 0.0408), *Cxcl10* (adjusted P = 0.0106), *Cd69* (adjusted P < 0.0001), and *Gzma* (adjusted P = 0.0161).

Together, these findings indicate that LysM-lineage *Hmgb1* deletion is associated with altered midbrain cytokine-related gene responses following systemic LPS exposure, with genotype-dependent differences observed for selected inflammatory transcripts at both 3 h and 7 days. We next examined whether LysM-lineage *Hmgb1* deletion was associated with altered immune gene expression and pathway responses in the cervical lymph nodes (CLNs) following LPS exposure.

### 3.4 CLN immune transcriptional responses following LPS exposure in mice with LysM-lineage *Hmgb1* deletion

To examine whether LysM-lineage Hmgb1 deletion was associated with altered immune transcriptional responses in the cervical lymph nodes (CLNs), gene expression was assessed at 3 h and 7 days following LPS or saline administration using the NanoString nCounter Mouse Immunology Panel (Fig. 4A–M). Genes with a Benjamini–Hochberg (BH)-adjusted P value < 0.05 were considered statistically significant, and log_2_ fold-change values were used to describe the direction and magnitude of gene-expression changes. Under saline-treated conditions, no significant differences in CLN gene expression were detected between *HMGB1*^*fl/fl*^ *LysM-Cre*-and *HMGB1*^*fl/fl*^ *LysM-Cre*+mice (data not shown).

**Fig. 4:**
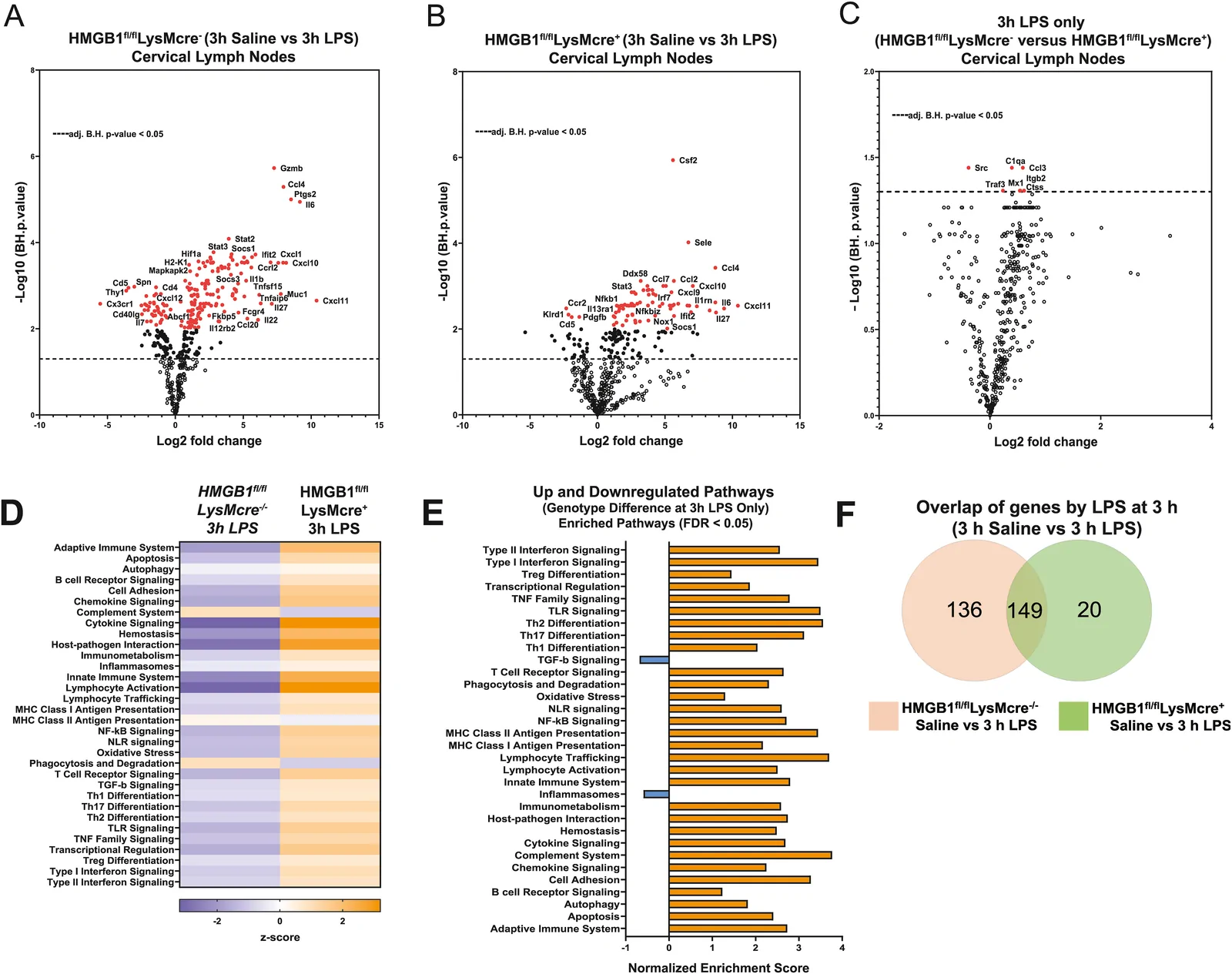

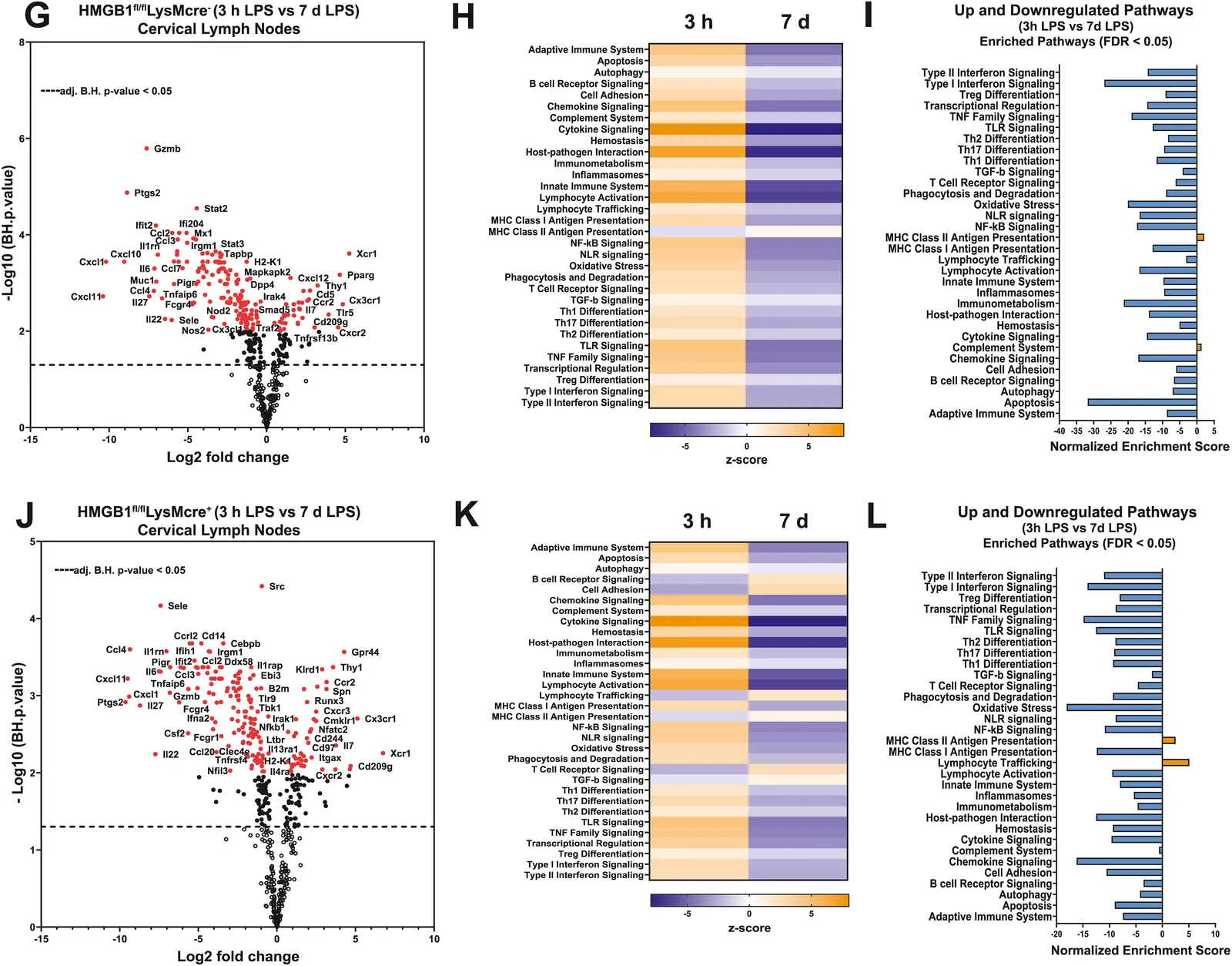

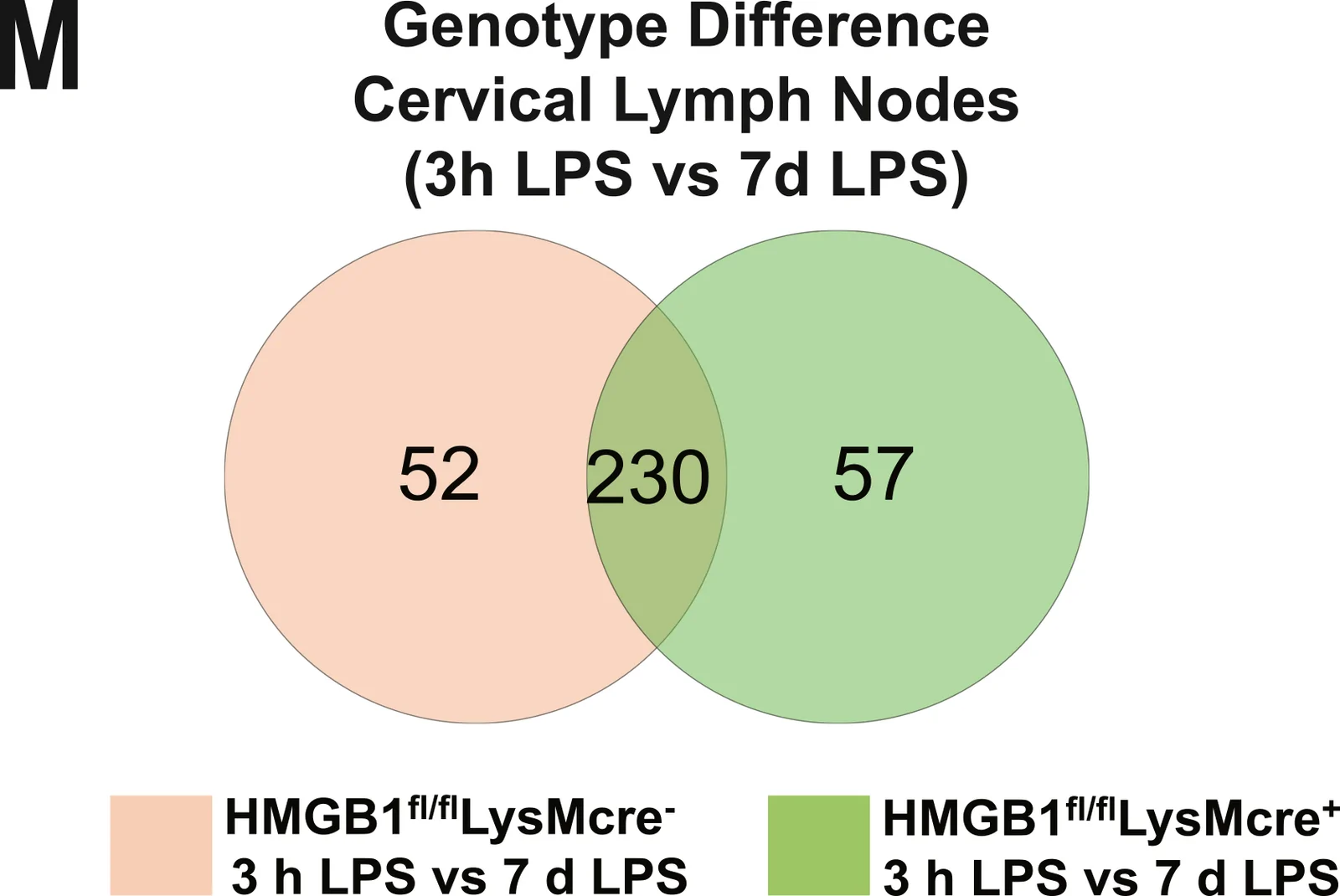
**Cervical lymph node immune transcriptional responses following systemic LPS exposure in mice with LysM-lineage *Hmgb1* deletion.** Male *HMGB1*^*fl/fl*^*LysM-cre*+mice and *HMGB1*^*fl/fl*^*LysM-cre*^-^ littermate controls received a single intraperitoneal injection of LPS (5 mg/kg) or saline. Cervical lymph nodes (CLNs) were collected at 3 h and 7 days after injection. Gene expression was assessed using the NanoString nCounter Mouse Immunology Panel, and differential gene-expression and pathway analyses were performed using nCounter Advanced Analysis. Volcano plots show differential gene expression at 3 h following LPS versus saline in *HMGB1*^*fl/fl*^*LysM-cre*-control mice (A) and *HMGB1*^*fl/fl*^*LysM-cre*+mice (B), and the direct genotype comparison between LPS-treated *HMGB1*^*fl/fl*^*LysM-cre*-and *HMGB1*^*fl/fl*^*LysM-cre*+mice at 3 h (C). Pathway z-score heatmap (D) and normalized enrichment score analysis (E) show immune pathway responses at 3 h; significantly enriched pathways in (E) met an FDR < 0.05. Venn diagram (F) shows the overlap of significantly regulated genes following LPS versus saline between genotypes at 3 h, with 149 genes shared between genotypes, 136 unique to *HMGB1*^*fl/fl*^*LysM-cre*-mice, and 20 unique to *HMGB1*^*fl/fl*^*LysM-cre*+mice. Volcano plots show differential gene expression between 3 h and 7 days following LPS in *HMGB1*^*fl/fl*^*LysM-cre*-control mice (G) and *HMGB1*^*fl/fl*^*LysM-cre*+mice (J). Corresponding pathway z-score heatmaps are shown in (H) and (K), and normalized enrichment score analyses are shown in (I) and (L); significantly enriched pathways met an FDR < 0.05. Venn diagram (M) shows the overlap of significantly differentially expressed genes between the 3-h and 7-day LPS comparisons in the two genotypes, with 230 genes shared between genotypes, 52 unique to *HMGB1*^*fl/fl*^*LysM-cre*^-^ mice, and 57 unique to *HMGB1*^*fl/fl*^*LysM-cre*+mice. For differential gene-expression analyses, genes with a Benjamini–Hochberg (BH)-adjusted *P* value < 0.05 were considered statistically significant. Log_2_ fold change indicates the direction and magnitude of gene-expression differences and was not applied as an additional significance threshold. Significantly differentially expressed genes are highlighted in red in the volcano plots, and the dashed horizontal line indicates the BH-adjusted *P* = 0.05 threshold. n = 3 biological samples per genotype × treatment group. Complete differential gene-expression and pathway-analysis results are provided in Supplementary Table S5A–S5E.

At 3 h following LPS exposure, both genotypes exhibited broad transcriptional responses relative to their respective saline-treated controls (Fig. 4A and B). Differential expression analysis identified 285 significantly regulated genes in *HMGB1*^*fl/fl*^ *LysM-Cre*-mice and 169 significantly regulated genes in *HMGB1*^*fl/fl*^ *LysM-Cre*+mice. Of these, 149 genes were shared between genotypes, whereas 136 genes were unique to *HMGB1*^*fl/fl*^ *LysM-Cre*^-^ mice and 20 genes were unique to *HMGB1*^*fl/fl*^ *LysM-Cre*+mice (Fig. 4F).

Direct comparison between LPS-treated genotypes at 3 h identified seven significantly differentially expressed genes based on BH-adjusted P < 0.05 (Fig. 4C). Relative to *HMGB1*^*fl/fl*^ *LysM-Cre*-mice, Src expression was lower, whereas *Traf3*, *C1qa*, *Ccl3*, *Mx1*, *Itgb2*, and *Ctss* expression was higher in *HMGB1*^*fl/fl*^ *LysM-Cre*+mice.

To evaluate functional patterns associated with these transcriptional responses, pathway enrichment analysis was performed using the NanoString Advanced Analysis module. Heatmap visualization showed genotype-associated differences in several immune-related pathway scores at 3 h following LPS exposure (Fig. 4D and E). Pathways related to the complement system, MHC class I antigen presentation, and phagocytosis showed lower pathway scores in *HMGB1*^*fl/fl*^ *LysM-Cre*+mice relative to *HMGB1*^*fl/fl*^ *LysM-Cre*-mice. TGF-β signaling and inflammasome-related pathways also showed lower normalized enrichment scores in *HMGB1*^*fl/fl*^ *LysM-Cre*+mice (FDR < 0.05).

Comparison of transcriptional responses between the 3-h and 7-day post-LPS time points identified time-dependent differences in both genotypes. In *HMGB1*^*fl/fl*^ *LysM-Cre*^-^ mice, 282 genes differed significantly between 3 h and 7 days, whereas 287 genes differed significantly between these time points in *HMGB1*^*fl/fl*^ *LysM-Cre* ^+^ mice. Of these, 230 genes were shared between genotypes, while 52 genes were unique to *HMGB1*^*fl/fl*^ *LysM-Cre*-mice and 57 genes were unique to *HMGB1*^*fl/fl*^ *LysM-Cre* ^+^ mice (Fig. 4M).

Pathway analysis of the temporal transcriptional responses identified changes in several immune-related pathways. In *HMGB1*^*fl/fl*^ *LysM-Cre*^-^ mice, MHC class II antigen presentation-related pathways differed between the 3-h and 7-day time points (Fig. 4H). In *HMGB1*^*fl/fl*^ *LysM-Cre* ^+^ mice, pathways related to B-cell receptor signaling, cell adhesion, lymphocyte trafficking, MHC class II antigen presentation, T-cell receptor signaling, and TGF-β signaling showed time-dependent differences (Fig. 4K). Normalized enrichment score analysis further identified enrichment of MHC class II antigen presentation and complement-related pathways in *HMGB1*^*fl/fl*^ *LysM-Cre*^-^ mice (Fig. 4I; FDR < 0.05) and lymphocyte trafficking and MHC class II antigen presentation-related pathways in *HMGB1*^*fl/fl*^ *LysM-Cre* ^+^ mice (Fig. 4L; FDR < 0.05).

Together, these findings indicate that LysM-lineage Hmgb1 deletion is associated with differences in CLN immune transcriptional responses following systemic LPS exposure, with genotype-associated differences evident at the 3-h time point and distinct temporal transcriptional patterns observed between 3 h and 7 days.

### 3.5 Spleen and liver transcriptional responses 7 days after LPS exposure in mice with LysM-lineage *Hmgb1* deletion

To examine transcriptional responses in peripheral immune organs during the later phase following systemic LPS exposure, gene expression was assessed in the spleen and liver 7 days after LPS or saline administration using the NanoString Mouse Inflammation Panel (Fig. 5 and Supplementary Fig. S2). Genes with a Benjamini–Hochberg (BH)-adjusted P value < 0.05 were considered statistically significant, and log_2_ fold-change values were used to describe the direction and magnitude of gene-expression changes.

**Fig. 5:**
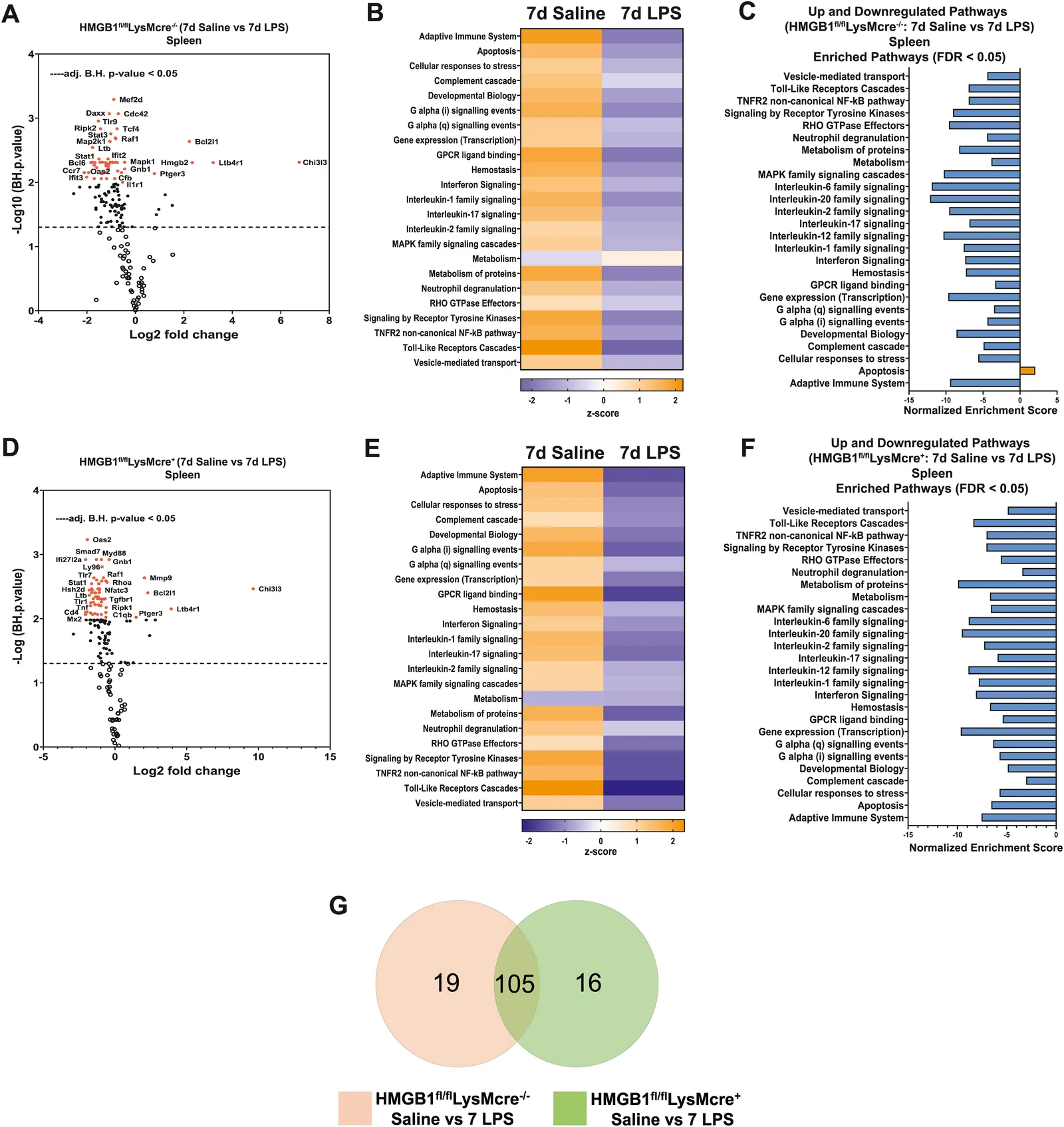
**Splenic inflammatory transcriptional responses 7 days after systemic LPS exposure in mice with LysM-lineage *Hmgb1* deletion.** Male *HMGB1*^*fl/fl*^*LysM-cre*^+^ and *HMGB1*^*fl/fl*^*LysM-cre*-(control) mice received a single intraperitoneal injection of LPS (5 mg/kg) or saline. Spleen tissues were collected at 7 days post-injection. Gene expression was assessed using the NanoString Mouse Inflammation Panel and analyzed with nCounter Advanced Analysis software. Volcano plots (A, D) depict differential gene expression following LPS relative to saline in *HMGB1*^*fl/fl*^*LysM-cre*-control mice (A) and *HMGB1*^*fl/fl*^*LysM-cre*+mice (D). Pathway z-score heatmaps (B, E) show pathway-level responses to saline and LPS treatment in *HMGB1*^*fl/fl*^*LysM-cre*-control mice (B) and *HMGB1*^*fl/fl*^*LysM-cre*+mice (E), with orange indicating positive and blue indicating negative z-scores. Pathway enrichment analyses (C, F) show normalized enrichment scores for significantly enriched pathways (FDR < 0.05) following LPS relative to saline in *HMGB1*^*fl/fl*^*LysM-cre*-control mice (C) and *HMGB1*^*fl/fl*^*LysM-cre*+mice (F). The Venn diagram (G) shows the overlap of genes significantly regulated by LPS relative to saline between genotypes, with 105 genes shared between genotypes, 19 unique to *HMGB1*^*fl/fl*^*LysM-cre*-mice, and 16 unique to *HMGB1*^*fl/fl*^*LysM-cre*+mice. Genes with a Benjamini–Hochberg-adjusted *P* value < 0.05 were considered significantly differentially expressed. Log_2_ fold change was used to indicate the direction and magnitude of gene-expression differences and was not applied as an additional significance threshold. n = 3 biological samples per genotype × treatment group. Complete differential gene-expression and pathway-analysis results are provided in Supplementary Table S6A–S6B.

In the spleen, differential expression analysis identified 140 genes significantly regulated by LPS across the two genotypes. Of these, 105 genes were shared between *HMGB1*^*fl/fl*^ *LysM-Cre*^-^ and *HMGB1*^*fl/fl*^ *LysM-Cre* ^+^ mice, whereas 19 genes were uniquely regulated in *HMGB1*^*fl/fl*^ *LysM-Cre*^-^ mice and 16 genes were uniquely regulated in *HMGB1*^*fl/fl*^ *LysM-Cre* ^+^ mice (Fig. 5A–D,G). Among the 16 genes uniquely regulated in *HMGB1*^*fl/fl*^ *LysM-Cre* ^+^ mice, four genes (*Shc1*, *Pla2g4a*, *Cysltr2*, and *Alox12*) were upregulated and 12 genes (*Ifit1*, *Il15*, *Tlr2*, *Ly96*, *Jun*, *Limk1*, *Tlr4*, *Gnaq*, *Max*, *Mknk1*, *Map2k4*, and *Myc*) were downregulated following LPS relative to saline. These included genes associated with lipid metabolism and inflammatory signaling, as well as pattern-recognition and cytokine signaling pathways. In *HMGB1*^*fl/fl*^ *LysM-Cre*^-^ mice, the 19 uniquely regulated genes (*Ccl24*, *Ccl22*, *Fasl*, *Nod2*, *Fxyd2*, *Tgfb3*, *C3*, *Ccl2*, *Pdgfa*, *Rock2*, *Ptk2*, *Tyrobp*, *Ptger2*, *Tgfb1*, *Mapkapk2*, *Prkca*, *C2*, *Il1r1*, and *Tollip*) were downregulated following LPS relative to saline and included genes associated with chemokine signaling, complement pathways, and intracellular signaling cascades.

Pathway enrichment analysis using the NanoString Advanced Analysis module identified changes in several spleen immune-related pathways following LPS exposure (Fig. 5B,C,E,F). Pathway z-score analysis showed changes in metabolism-related and other immune-associated pathways between saline- and LPS-treated mice within each genotype. Normalized enrichment score analysis identified significantly enriched pathways in both genotypes (FDR < 0.05; Fig. 5C–F).

In the liver, transcriptional changes were limited at 7 days following LPS exposure. In *HMGB1*^*fl/fl*^ *LysM-Cre*^-^ mice, *Gnb1* was significantly downregulated following LPS compared with saline (log_2_FC = −0.660, BH-adjusted P = 0.0148). No genes met the BH-adjusted P < 0.05 significance criterion following LPS in *HMGB1*^*fl/fl*^ *LysM-Cre* ^+^ mice (Supplementary Fig. S2; Supplementary Table S6C–D).

Together, these findings demonstrate substantial overlap in the splenic transcriptional response to LPS between genotypes at 7 days, with smaller subsets of genes uniquely regulated within each genotype, whereas transcriptional changes in the liver were limited. We next examined CD45 immunoreactivity at the choroid plexus to assess inflammatory responses at a CNS interface following systemic LPS exposure.

### 3.6 Choroid plexus CD45 immunoreactivity following LPS exposure in mice with LysM-lineage *Hmgb1* deletion

The choroid plexus forms the blood–cerebrospinal fluid barrier and contributes to immune communication between the periphery and central nervous system (CNS) (Javed et al., 2026). To determine whether LysM-lineage Hmgb1 deletion was associated with altered CD45 immunoreactivity at this CNS interface, brains were collected from *HMGB1*^*fl/fl*^ *LysM-Cre*^-^ and *HMGB1*^*fl/fl*^ *LysM-Cre*+mice at 3 h and 7 days following LPS or saline administration (Fig. 6). Immunofluorescence staining for CD45 was performed on sagittal brain sections containing the lateral ventricle choroid plexus, and CD45 fluorescence intensity was quantified within defined choroid plexus regions of interest.

**Fig. 6:**
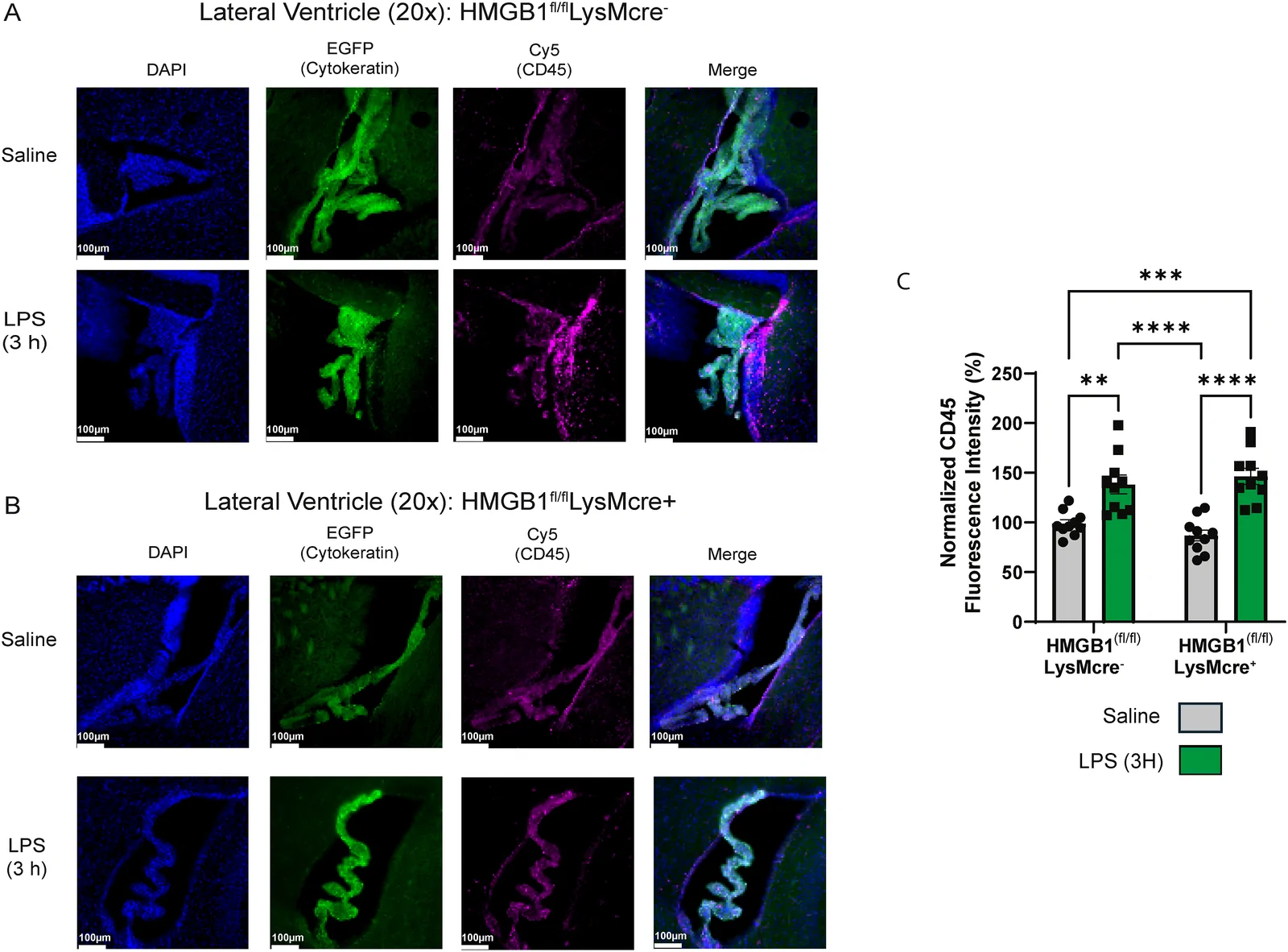

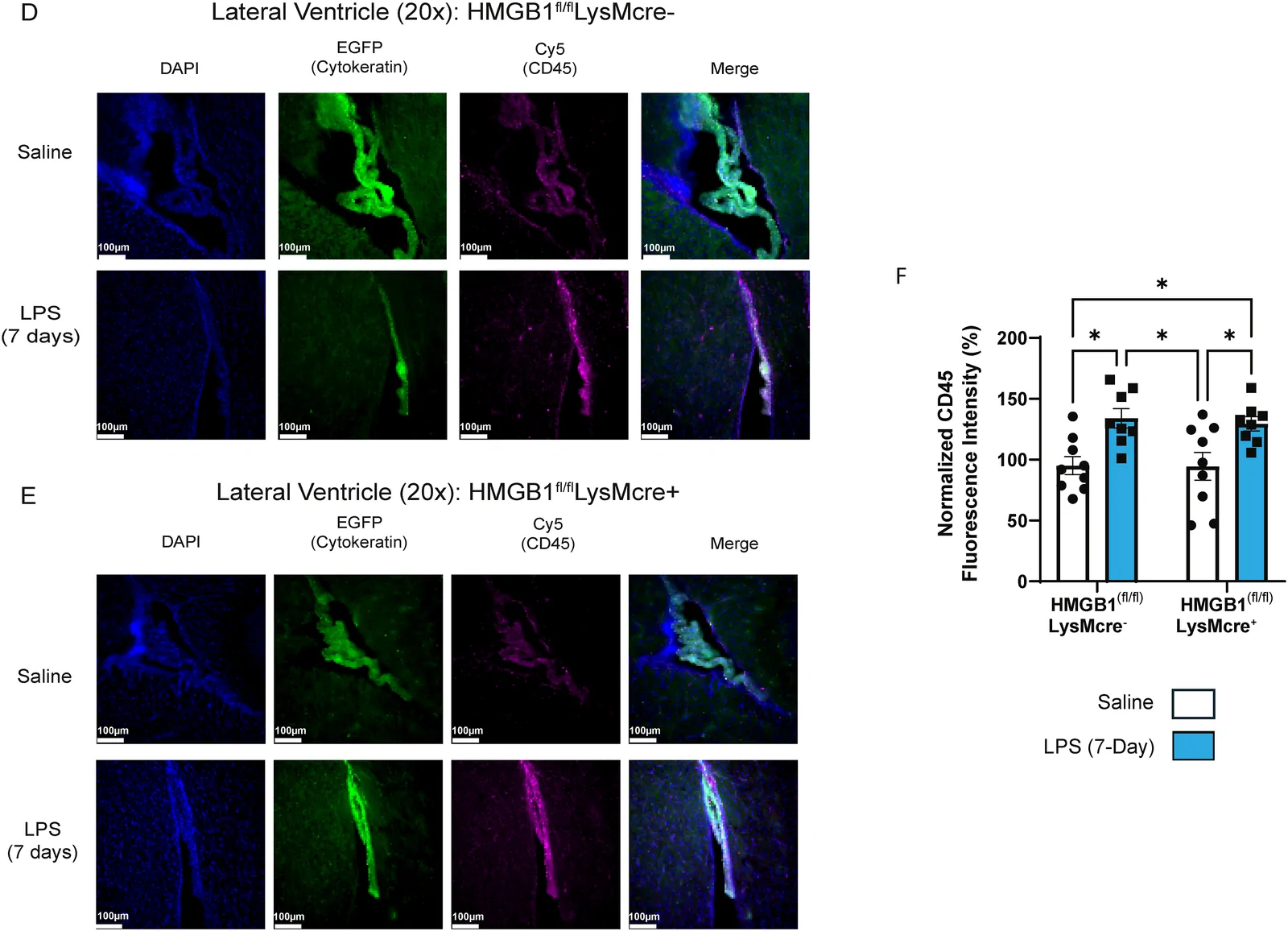
**Choroid plexus CD45 immunoreactivity following systemic LPS exposure in mice with LysM-lineage *Hmgb1* deletion.** Male *HMGB1*^*fl/fl*^*LysM-cre*+and *HMGB1*^*fl/fl*^*LysM-cre*-littermate control mice received a single intraperitoneal injection of LPS (5 mg/kg) or saline. Brain tissue was collected at 3 h (acute; A–C) and 7 days (later post-exposure; D–F) after injection. Immunofluorescence staining for CD45 was performed on sagittal brain sections containing the lateral ventricle choroid plexus. CD45 fluorescence intensity was quantified within defined choroid plexus regions of interest following background subtraction. Representative immunofluorescence images of the lateral ventricle choroid plexus are shown at 20× magnification for each genotype and time point (3 h: A, B; 7 days: D, E), with corresponding quantification shown in C and F. Negative-control sections processed without primary antibodies but with the corresponding secondary antibodies and DAPI showed no detectable CD45-specific signal. Data are presented as mean ± SEM (n = 10 mice per group at 3 h; n = 8–9 mice per group at 7 days). Statistical analysis was performed using two-way ANOVA with genotype and treatment as factors, followed by Bonferroni post hoc multiple comparisons. LPS produced a significant main effect of treatment at both 3 h and 7 days, whereas no significant main effect of genotype or genotype × treatment interaction was detected at either time point. CD45 fluorescence intensity did not differ significantly between genotypes following LPS at either time point. *P < 0.05, **P < 0.01, ***P < 0.001, ****P < 0.0001. Complete statistical results are provided in Supplementary Table S7A–S7C.

At 3 h post-LPS, two-way ANOVA revealed a significant main effect of treatment on CD45 fluorescence intensity (F(1,36) = 49.36, P < 0.0001), with no significant main effect of genotype (F(1,36) = 0.06489, P = 0.8004) or genotype × treatment interaction (F(1,36) = 2.046, P = 0.1612). Bonferroni post hoc comparisons showed significantly higher CD45 fluorescence intensity following LPS compared with saline in both *HMGB1*^*fl/fl*^ *LysM-Cre*-mice (adjusted P = 0.0021) and *HMGB1*^*fl/fl*^ *LysM-Cre* ^+^ mice (adjusted P < 0.0001). CD45 fluorescence intensity did not differ significantly between genotypes following LPS (adjusted P > 0.9999; Fig. 6A–C).

At 7 days post-LPS, two-way ANOVA also revealed a significant main effect of treatment on CD45 fluorescence intensity (F(1,30) = 18.61, P = 0.0002), with no significant main effect of genotype (F(1,30) = 0.08534, P = 0.7722) or genotype × treatment interaction (F(1,30) = 0.05008, P = 0.8244). Bonferroni post hoc comparisons showed significantly higher CD45 fluorescence intensity following LPS compared with saline in both *HMGB1*^*fl/fl*^ *LysM-Cre*-mice (adjusted P = 0.0158) and *HMGB1*^*fl/fl*^ *LysM-Cre* ^+^ mice (adjusted P = 0.0337). CD45 fluorescence intensity did not differ significantly between genotypes following LPS (adjusted P = 0.9844; Fig. 6D–F).

Together, these findings indicate that systemic LPS exposure was associated with increased choroid plexus CD45 immunoreactivity at both 3 h and 7 days, whereas LysM-lineage Hmgb1 deletion was not associated with a significant difference in this response at either time point.

### 3.7 Bone marrow transcriptional responses 7 days after LPS exposure in mice with LysM-lineage *Hmgb1* deletion

To assess bone marrow transcriptional responses in mice with LysM-lineage Hmgb1 deletion, RNA was isolated from the bone marrow of *HMGB1*^*fl/fl*^ *LysM-Cre*^-^ and *HMGB1*^*fl/fl*^ *LysM-Cre* ^+^ mice 7 days after LPS or saline administration and analyzed using the NanoString Mouse Immunology Panel (Fig. 7). Genes with a Benjamini–Hochberg (BH)-adjusted P value < 0.05 were considered statistically significant, and log_2_ fold-change values were used to describe the direction and magnitude of gene-expression changes.

**Fig. 7:**
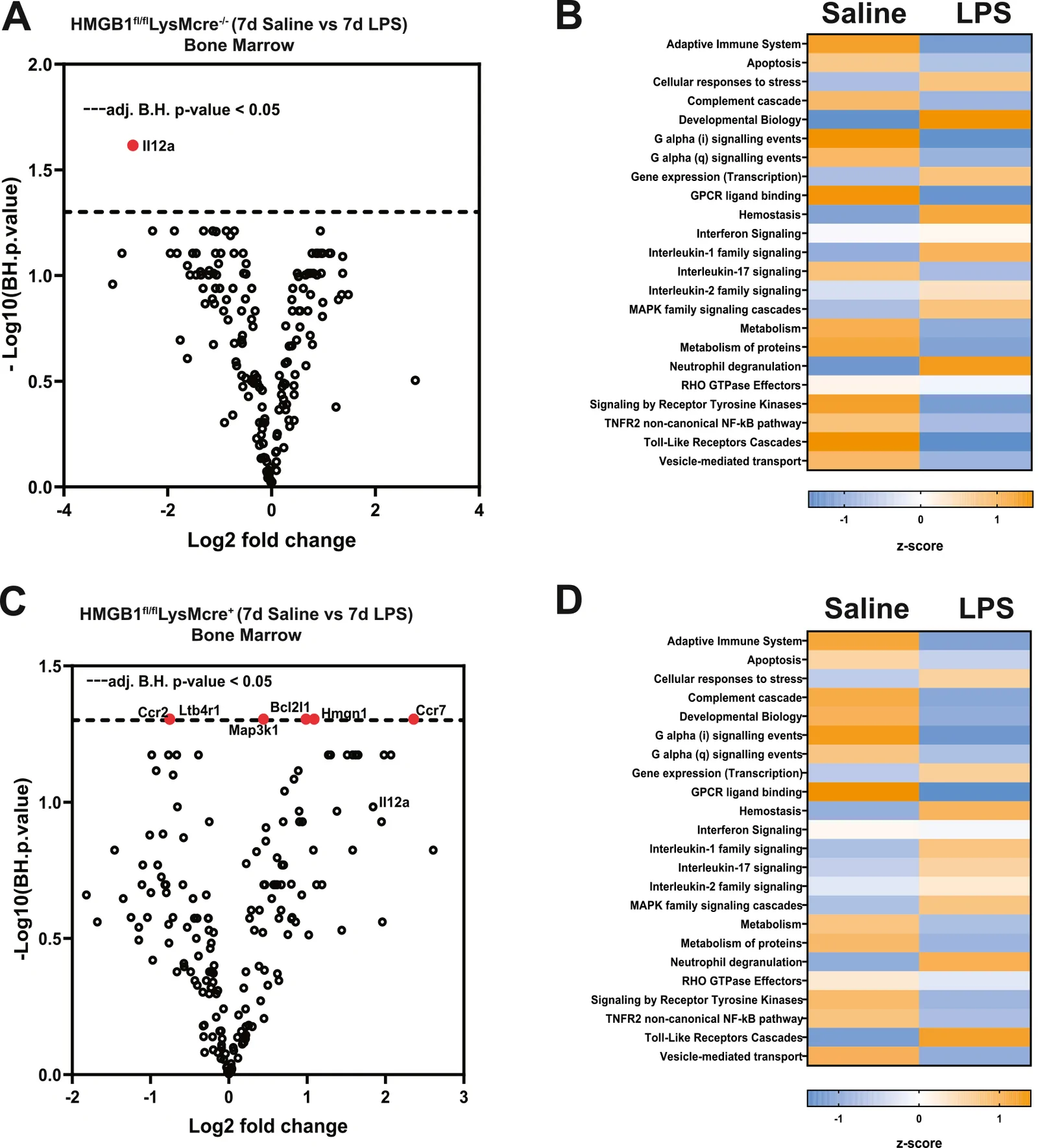
**Bone marrow transcriptional responses 7 days after LPS exposure in mice with LysM-lineage *Hmgb1* deletion.** Male *HMGB1*^*fl/fl*^*LysM-cre*+mice and *HMGB1*^*fl/fl*^*LysM-cre*-littermate controls received a single intraperitoneal injection of LPS (5 mg/kg) or saline, and bone marrow was collected 7 days after injection. Gene expression was assessed using the NanoString nCounter Mouse Immunology Panel and analyzed using the nCounter Advanced Analysis module. **(A, C)** Volcano plots show differential gene expression comparing LPS versus saline within *HMGB1*^*fl/fl*^*LysM-cre*-control mice (A) and *HMGB1*^*fl/fl*^*LysM-cre*+mice (C). Log_2_ fold change is shown on the x-axis and −log_10_(BH-adjusted *P* value) on the y-axis. The horizontal reference line at **1.301 corresponds to a BH-adjusted *P* value of 0.05**; genes above this threshold were considered significantly differentially expressed. Log_2_ fold change indicates the direction and magnitude of gene-expression changes and was not applied as an additional significance threshold. Significantly differentially expressed genes are highlighted in red. **(B, D)** Pathway z-score heatmaps show pathway-level responses following saline or LPS treatment in *HMGB1*^*fl/fl*^*LysM-cre*-control mice (B) and *HMGB1*^*fl/fl*^*LysM-cre*+mice (D), with pathway z-scores represented according to the indicated color scale. n = 3 independent biological samples per genotype × treatment group**.** Complete differential gene-expression and pathway-analysis results are provided in Supplementary Table S8A–S8B.

Pathway z-score analysis showed changes in several immune- and stress-related pathways following LPS exposure in both genotypes, including interleukin signaling, neutrophil degranulation, MAPK signaling, and cellular stress-response pathways (Fig. 7B–D).

Gene-level analysis identified seven significantly regulated genes across the two within-genotype LPS-versus-saline comparisons (Fig. 7A–C). In *HMGB1*^*fl/fl*^ *LysM-Cre*^-^ mice, Il12a was downregulated following LPS relative to saline. In *HMGB1*^*fl/fl*^ *LysM-Cre* ^+^ mice, *Ccr2* and *Ltb4r1* were downregulated, whereas *Map3k1*, *Bcl2l1*, *Hmgn1*, and *Ccr7* were upregulated following LPS relative to saline.

Together, these findings indicate that relatively few bone marrow genes met the statistical significance criterion 7 days after LPS exposure in either genotype, while pathway z-score analyses showed changes in several immune- and stress-related pathways.

## 4 Discussion

### 4.1 LysM-lineage *Hmgb1* deletion is associated with compartment-specific inflammatory responses following systemic LPS exposure

The present study examined inflammatory responses across peripheral and central compartments following systemic LPS exposure in mice with LysM-lineage *Hmgb1* deletion. A central finding was that LPS increased circulating HMGB1 in both genotypes, whereas total plasma HMGB1 concentrations did not differ significantly between genotypes at either 3 h or 7 days. Despite comparable circulating HMGB1 concentrations, distinct genotype-associated transcriptional responses were detected in the midbrain and cervical lymph nodes (CLNs). In comparison, later transcriptional responses in the spleen showed substantial overlap between genotypes, while gene-level responses in the liver and bone marrow were more limited. These findings demonstrate that inflammatory responses associated with LysM-lineage *Hmgb1* deletion vary across anatomical compartments following systemic LPS challenge.

The present study used a single 5 mg/kg LPS challenge as a controlled model of experimental endotoxemia. This sublethal challenge produces robust systemic inflammation and reproduces selected features of sepsis-associated inflammatory responses (Fernandez-Martin et al., 2022; Fink, 2014; Lewis et al., 2016; Seemann et al., 2017). The 3-h time point captured acute inflammatory responses, whereas the 7-day time point provided an opportunity to examine later responses following the initial systemic challenge. The presence of inflammatory transcriptional and immunoreactivity changes at 7 days indicates that selected immune-related responses remained detectable well beyond the acute time point. Because responses were not measured between 3 h and 7 days, these findings are best described as later post-LPS responses and do not establish the intervening inflammatory trajectory.

HMGB1 is a nuclear DNA-binding protein that can function extracellularly as a damage-associated molecular pattern (DAMP), activating inflammatory pathways through receptors including TLR4 and RAGE (Andersson and Tracey, 2011; Tang et al., 2011; Hreggvidsdottir et al., 2012). LPS exposure increased total plasma HMGB1 concentrations in both genotypes, while LysM-lineage *Hmgb1* deletion did not significantly alter circulating HMGB1 at either measured time point. Circulating HMGB1 can originate from multiple cellular sources during systemic inflammation, including hepatocytes, endothelial cells, granulocytes, and damaged tissues (Andersson and Tracey, 2003; Deng et al., 2019). The maintenance of circulating HMGB1 following LysM-lineage *Hmgb1* deletion is therefore consistent with a systemic HMGB1 pool to which multiple cellular populations may contribute. Moreover, active HMGB1 release following endotoxin stimulation has been described as a delayed response relative to early inflammatory cytokines (Bonaldi et al., 2003; Gardella et al., 2002; Scaffidi et al., 2002; Wang et al., 1999). Thus, HMGB1 detected at 3 h may reflect contributions from pre-existing intracellular HMGB1 and other early sources in addition to newly synthesized and released protein. These findings highlight the complexity of the circulating HMGB1 response and suggest that total plasma concentration alone may not reflect the contribution of individual cellular sources.

A notable finding was the presence of genotype-associated differences in selected midbrain inflammatory transcripts despite comparable circulating HMGB1 concentrations. At 3 h, differences were observed in *Il1b, Tnf,* and *Cd69*, while at 7 days differences were detected across several inflammatory transcripts, including *Il1b, Tnf, Cxcl10, Cd69,* and *Gzma*. These results demonstrate that LysM-lineage *Hmgb1* deletion is associated with altered central inflammatory transcriptional responses following systemic LPS exposure. The dissociation between total circulating HMGB1 and tissue-level transcriptional responses further suggests that local tissue context, cellular interactions, receptor availability, and other inflammatory mediators may contribute to the compartment-specific effects associated with HMGB1 signaling.

The CLNs also showed prominent transcriptional responses associated with LysM-lineage *Hmgb1* deletion. At 3 h, both genotypes exhibited broad responses to LPS, with a large set of shared significantly regulated genes together with smaller genotype-specific subsets. Direct comparison of LPS-treated genotypes further identified a limited group of differentially expressed genes. Pathway analyses identified differences involving antigen presentation, complement, phagocytosis, TGF-β signaling, and inflammasome-related pathways. In addition, comparisons between the 3-h and 7-day time points revealed substantial temporal changes in CLN transcriptional responses in both genotypes. These findings identify the CLNs as an immune compartment responsive to systemic LPS exposure in which LysM-lineage *Hmgb1* deletion is associated with distinct transcriptional features. Given the established relationship between CNS-associated lymphatic drainage and cervical lymph nodes (Ahn et al., 2019; Aspelund et al., 2015; Louveau et al., 2015), these observations provide a basis for future studies examining how CLN responses relate to central inflammatory signaling.

The responses observed in other peripheral compartments further illustrate the tissue specificity of the systemic LPS response. At 7 days, splenic transcriptional responses showed substantial overlap between genotypes, together with smaller subsets of genes regulated within the individual genotype comparisons. Liver transcriptional changes were limited, while relatively few individual bone marrow genes reached statistical significance despite changes in several immune- and stress-related pathway scores. Thus, the transcriptional consequences of systemic LPS exposure at the later time point differed substantially among peripheral immune compartments. The contrast between these patterns and the genotype-associated responses observed in the midbrain and CLNs emphasizes the importance of anatomical and cellular context in shaping inflammatory responses associated with LysM-lineage *Hmgb1* deletion.

The choroid plexus showed a robust response to systemic LPS exposure, with increased CD45 immunoreactivity at both 3 h and 7 days. However, CD45 fluorescence intensity did not differ significantly between genotypes following LPS exposure at either time point. These findings indicate that the choroid plexus remained responsive to systemic inflammatory challenge at the later time point, while LysM-lineage *Hmgb1* deletion was not associated with a detectable difference in CD45 immunoreactivity. Because the analysis quantified fluorescence intensity within defined choroid plexus regions of interest, these data reflect CD45 immunoreactivity rather than direct enumeration of infiltrating or resident immune cells.

The conditional model used in this study has previously been characterized as showing reduced *Hmgb1* expression in peripheral LysM-expressing macrophage populations without corresponding changes in brain or microglial *Hmgb1* expression (Greve et al., 2023; Ahmed et al., 2024; Yamada et al., 2026). LysM-Cre-mediated recombination is prominent in monocyte/macrophage and granulocyte lineages (Clausen et al., 1999), providing a means of examining the contribution of these LysM-expressing populations to inflammatory responses following systemic challenge. The present findings therefore extend previous characterization of this model by demonstrating that LysM-lineage *Hmgb1* deletion is associated with distinct responses across peripheral and central inflammatory compartments.

Taken together, the multi-compartment analysis is an important feature of the present study. Examination of plasma, midbrain, CLNs, spleen, liver, bone marrow, and the choroid plexus revealed that the inflammatory responses associated with LysM-lineage *Hmgb1* deletion were not uniform across tissues (Fig. 8). The most prominent genotype-associated transcriptional differences were detected in the midbrain and CLNs despite comparable circulating HMGB1 concentrations. These findings support a model in which inflammatory responses associated with LysM-lineage Hmgb1 deletion are influenced by tissue context following systemic LPS exposure.

**Fig. 8:**
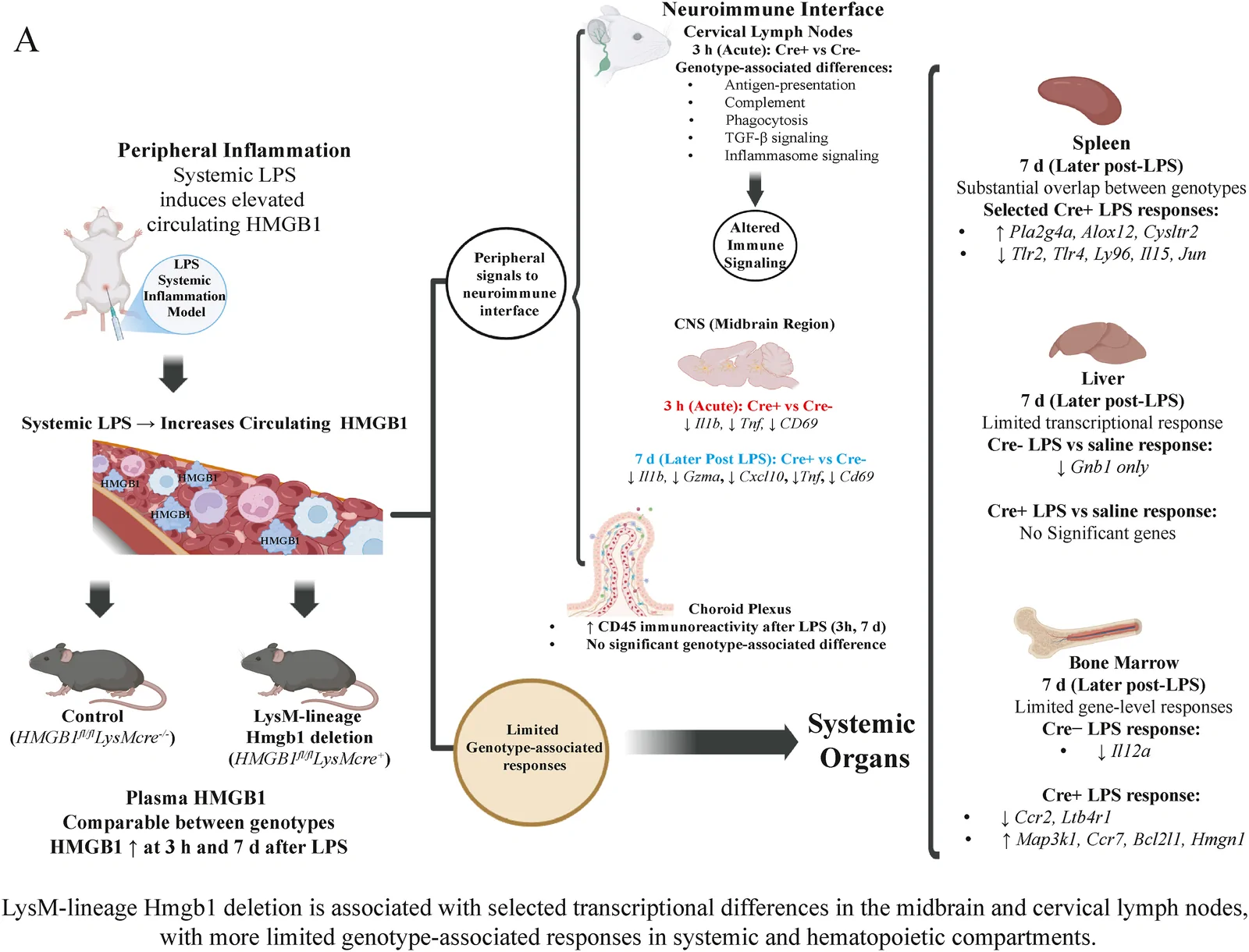
**Conceptual summary of compartment-specific inflammatory responses following systemic LPS exposure in mice with LysM-lineage *Hmgb1* deletion.** Systemic LPS exposure increases circulating HMGB1 at 3 h and 7 days, with comparable plasma HMGB1 concentrations between genotypes. Despite similar circulating HMGB1 concentrations, LysM-lineage *Hmgb1* deletion is associated with genotype-dependent inflammatory transcriptional responses in the midbrain and cervical lymph nodes. In the midbrain, genotype-associated differences in selected inflammatory transcripts were detected at both 3 h and 7 days. Cervical lymph nodes exhibit genotype-associated transcriptional and immune-pathway differences at 3 h. Systemic LPS also increases choroid plexus CD45 immunoreactivity at 3 h and 7 days without a significant genotype-associated difference. At 7 days, splenic transcriptional responses show substantial overlap between genotypes, whereas gene-level responses in the liver and bone marrow are comparatively limited. Together, these findings support compartment- and time-dependent inflammatory responses associated with LysM-lineage *Hmgb1* deletion following systemic LPS exposure.

### 4.2 Behavioral considerations

Behavioral testing revealed no significant differences in spontaneous alternation or locomotor activity in the Y-maze at 7 days following LPS exposure. This assessment was performed at the later post-LPS time point rather than during the acute period, when systemic LPS produces prominent sickness-related behavioral changes (Lasselin et al., 2020; Matsuura et al., 2023; Arai et al., 2001). The behavioral findings therefore indicate that differences in Y-maze performance were not detectable at 7 days, despite genotype-associated differences in selected inflammatory transcriptional responses.

The timing of behavioral assessment is important for interpreting these results. Behavioral testing was not performed during the acute response or at intermediate post-LPS time points. Consequently, transient differences during recovery may not have been captured by the 7-day assessment. Behavioral evaluation during the intermediate 2–4-day period could help define the relationship between inflammatory recovery and behavioral outcomes and determine whether molecular differences are accompanied by transient changes in behavioral performance.

Animal age may also influence responses to systemic inflammatory challenge. Mice were 7–8 weeks old at the time of LPS administration, representing a relatively young age. Age-dependent differences in susceptibility and inflammatory cytokine responses to systemic LPS have been reported in mice (Tateda et al., 1996), and future studies across broader age ranges could determine whether the molecular and behavioral responses observed here are maintained in older animals.

Y-maze testing was performed between 09:00 and 13:00 h during the light phase. Although all groups were tested under identical conditions, mice are nocturnal and exploratory activity can vary with diurnal phase (Tsao et al., 2022). Behavioral assessments during the active phase and across additional post-LPS time points may therefore provide further information regarding the temporal relationship between inflammatory and behavioral responses.

Overall, the behavioral and molecular findings provide complementary information. No differences in spontaneous alternation or locomotor activity were detected at the 7-day assessment, whereas selected inflammatory transcriptional responses differed between genotypes. Thus, the molecular responses associated with LysM-lineage *Hmgb1* deletion were detectable in the absence of a corresponding Y-maze phenotype at the later time point examined.

### 4.3 Limitations

Several considerations define the scope of the present findings and provide opportunities for further investigation. The conditional *Hmgb1* model used in this study has been previously characterized (Greve et al., 2023; Ahmed et al., 2024), and experimental animals were genotyped by PCR to confirm the floxed *Hmgb1* and LysM-Cre alleles. Direct quantification of HMGB1 depletion in isolated LysM-expressing peripheral populations was not performed in the present study. Future assessment of HMGB1 expression in purified LysM-targeted populations under baseline and LPS-stimulated conditions would further strengthen cell-specific characterization of the model and help relate deletion efficiency to the compartment-specific responses identified here.

LysM-Cre-mediated recombination is prominent in monocyte/macrophage and granulocyte lineages but does not uniformly encompass all populations conventionally classified as myeloid (Clausen et al., 1999). The present findings therefore specifically reflect the LysM-lineage genetic manipulation. Complementary cell-specific approaches could further distinguish the relative contributions of individual peripheral immune populations to the responses observed across tissues.

Total plasma HMGB1 measurements provide an integrated measure of the circulating HMGB1 pool but do not distinguish its cellular sources. Comparable plasma HMGB1 concentrations between genotypes demonstrate that LysM-lineage *Hmgb1* deletion was not sufficient to significantly reduce total circulating HMGB1 under the conditions examined, while contributions from LysM-lineage and other HMGB1-producing populations remain possible. In addition, the 3-h measurement may include release of pre-existing intracellular HMGB1 as well as newly synthesized and released protein. Cell-specific and temporally resolved measurements would further define the cellular contributions to circulating HMGB1 following systemic LPS exposure.

The inclusion of acute (3 h) and later (7-day) time points enabled comparison of inflammatory responses at two distinct stages following systemic LPS challenge. Intermediate time points were not examined and would provide additional resolution of the transition between these stages. Such analyses could determine whether transcriptional responses detected at 7 days were continuously sustained or changed during the intervening period and could identify transient behavioral or molecular responses during recovery.

The mice were 7–8 weeks old at LPS administration, and inflammatory susceptibility and recovery may vary with age. Similarly, Y-maze testing was conducted during the light phase. Although age and behavioral testing conditions were standardized across experimental groups, studies incorporating broader age ranges and behavioral testing during the active phase would further establish the generalizability of the findings.

Only male mice were examined in the present study. Inclusion of both sexes in future studies will be important for determining whether sex influences HMGB1-associated peripheral and central inflammatory responses.

The multi-compartment transcriptional approach enabled comparison of immune-related responses across several peripheral and central tissues. Additional protein-level and functional analyses would further establish the biological consequences of the transcriptional differences identified here. Similarly, CD45 analysis quantified fluorescence intensity within choroid plexus regions of interest; cell-resolved approaches could further determine which immune-cell populations contribute to these changes in immunoreactivity.

The NanoString panels provided targeted profiling of immune- and inflammation-related genes across multiple tissues with correction for multiple testing using Benjamini–Hochberg-adjusted P values. Because these panels contain predefined gene sets, unbiased transcriptomic approaches may identify additional pathways not represented by the panels.

Finally, the 5 mg/kg LPS challenge provides a controlled, sublethal model of experimental endotoxemia that produces robust systemic inflammatory signaling and selected features of sepsis-associated inflammation. Complementary inflammatory models will be useful for determining how broadly the compartment-specific responses identified here extend across different systemic inflammatory conditions.

### 4.4 Future directions

The compartment-specific responses identified in this study provide several directions for further mechanistic investigation. Direct quantification of HMGB1 in isolated LysM-expressing peripheral populations under baseline and LPS-stimulated conditions would further define the cellular specificity and efficiency of conditional *Hmgb1* deletion. Complementary cell-specific approaches could distinguish the relative contributions of monocyte/macrophage, granulocyte, and other immune populations to the responses observed following systemic LPS exposure.

Identifying the cellular sources contributing to circulating HMGB1 will also help clarify how total plasma HMGB1 is maintained following LysM-lineage *Hmgb1* deletion. Cell-specific measurements of HMGB1 release and temporally resolved plasma analyses could distinguish contributions from pre-existing intracellular HMGB1 release and newly synthesized protein.

Single-cell and spatial transcriptomic approaches could identify the cellular populations underlying the transcriptional differences observed in the midbrain and CLNs. Complementary proteomic, cytokine, and receptor-signaling analyses could determine whether these transcriptional responses translate to corresponding functional changes and further define the involvement of HMGB1-associated signaling through receptors such as TLR4 and RAGE.

Additional temporal studies incorporating intermediate post-LPS time points will be particularly useful for defining the progression from acute systemic inflammation to the later responses detected at 7 days. Combining these measurements with behavioral assessments during recovery could determine whether transient behavioral responses accompany the molecular changes identified here. Studies incorporating both sexes, broader age ranges, and behavioral testing during the active phase would further establish the generalizability of these findings.

## 5 Conclusion

In summary, the present study demonstrates that LysM-lineage *Hmgb1* deletion is associated with compartment-specific inflammatory responses following systemic LPS exposure. LPS increased total circulating HMGB1 concentrations at both 3 h and 7 days, but these concentrations did not differ significantly between genotypes. In contrast, selected inflammatory transcriptional responses in the midbrain and CLNs showed clear genotype-associated differences, demonstrating that tissue-specific inflammatory responses can differ despite comparable total circulating HMGB1 concentrations.

The multi-compartment analysis further demonstrated that these responses were not uniform across tissues. Splenic transcriptional responses at 7 days showed substantial overlap between genotypes, gene-level responses in the liver and bone marrow were comparatively limited, and systemic LPS increased choroid plexus CD45 immunoreactivity without a significant genotype-associated difference. Together, these findings identify the midbrain and CLNs as prominent compartments in which inflammatory transcriptional responses were associated with LysM-lineage *Hmgb1* deletion following systemic LPS challenge.

The maintenance of circulating HMGB1 following LysM-lineage *Hmgb1* deletion further highlights the complexity of the systemic HMGB1 response and is consistent with contributions from multiple cellular sources. At the same time, the distinct midbrain and CLN transcriptional responses demonstrate that total circulating HMGB1 concentration does not fully capture the compartment-specific inflammatory responses associated with the LysM-lineage genetic manipulation.

Overall, these findings establish tissue context as an important feature of HMGB1-associated inflammatory responses following systemic LPS exposure (Fig. 8). The identification of distinct midbrain and CLN transcriptional responses in the presence of comparable circulating HMGB1 provides a foundation for defining how cellular source, anatomical compartment, and local signaling environments shape inflammatory responses following systemic immune activation.

## CRediT authorship contribution statement

**James A. Johnson:** Writing – review & editing, Writing – original draft, Visualization, Supervision, Project administration, Investigation, Formal analysis, Data curation, Conceptualization. **Angar Tsoggerel:** Writing – review & editing, Validation, Investigation, Formal analysis. **Kathryn Fischer:** Writing – review & editing, Methodology, Investigation, Formal analysis. **Alexandru Movila:** Writing – review & editing, Methodology, Investigation. **Antentor Hinton:** Writing – review & editing. **Kimberly Sullivan:** Writing – review & editing, Methodology, Investigation.

## Ethics declaration

This study was conducted in accordance with the ARRIVE (Animal Research: Reporting of In Vivo Experiments) guidelines. This study was approved by the Indiana University School of Medicine Institutional Animal Care and Use Committee. (Approval No. 24046 and 20092)

## Declaration of competing interest

All authors have no known competing financial interests or personal relationships that could have appeared to influence the work reported in this paper.

## Data Availability

Data will be made available on request.
