# Supplementary material for: Tissue-specific inflammatory responses to systemic LPS in mice with LysM-lineage Hmgb1 deletion
Source: Brain Behav Immun Health. 2026 Oct 6;58:101382. doi: 10.1016/j.bbih.2026.101382 (PMC13677236; doi:10.1016/j.bbih.2026.101382)
Supplement: Supplementary file 1 [file mmc8.pdf]

A.

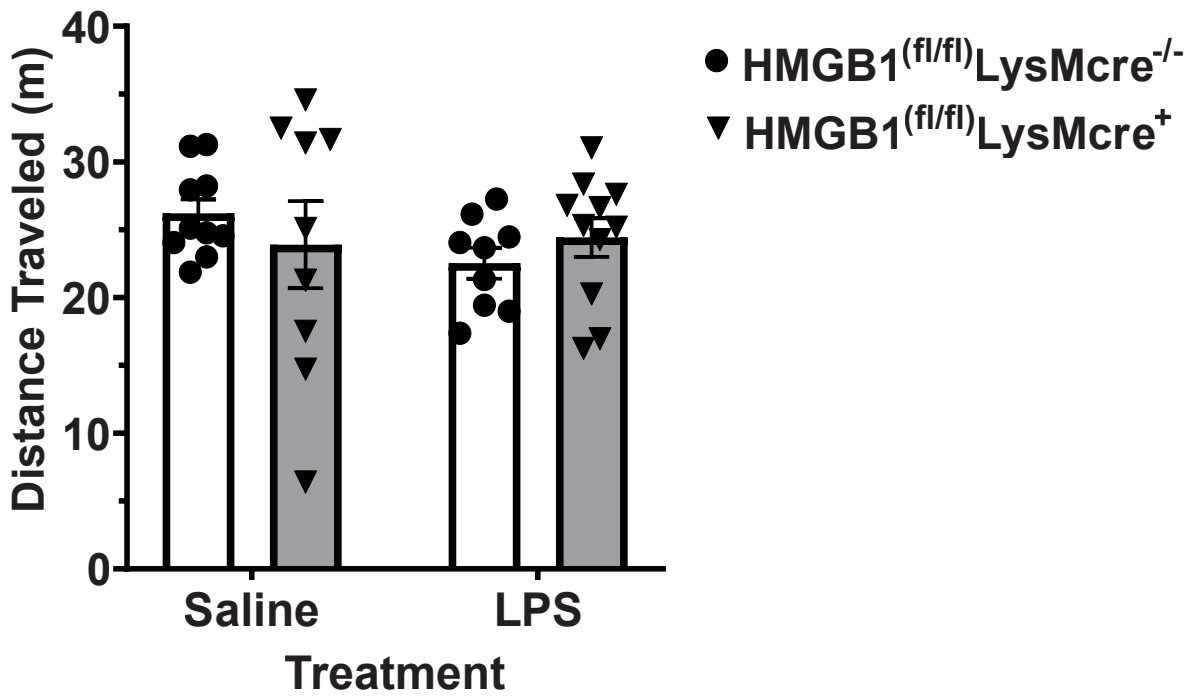

40
30
20
10
0
Distance Traveled (m)
Saline
LPS
Treatment
HMGB1(fl/fl)LysMcre-/-
HMGB1(fl/fl)LysMcre+

B.

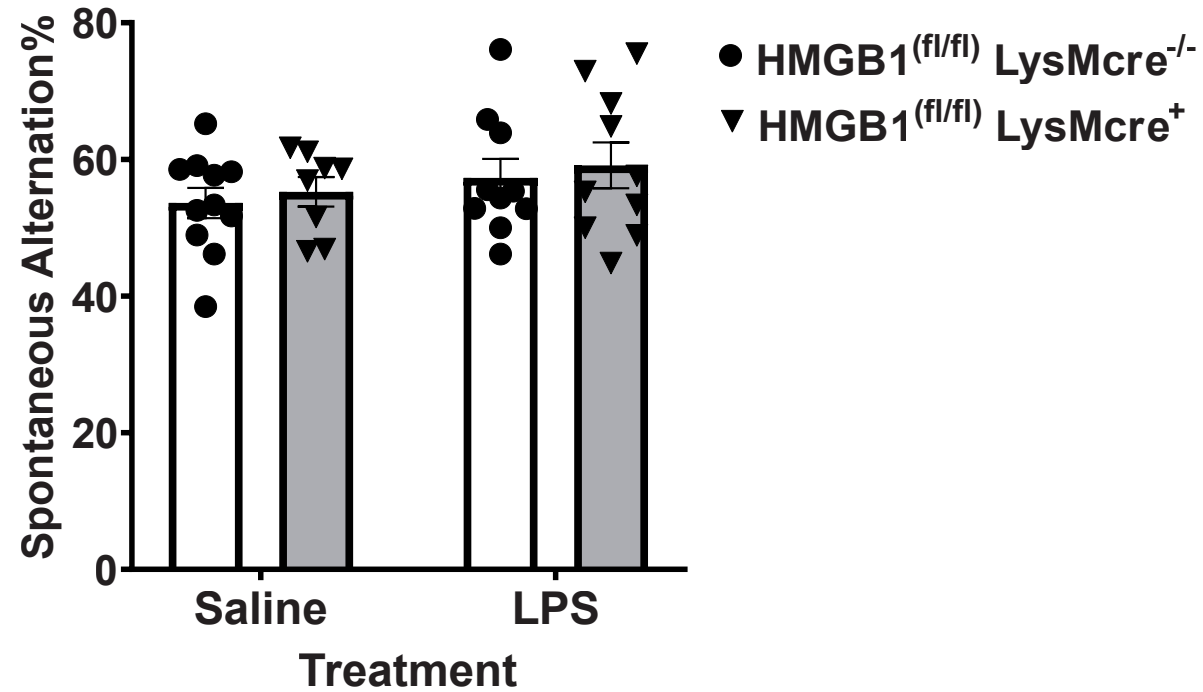

80
60
40
20
0
Spontaneous Alternation%
Saline
LPS
Treatment
HMGB1(fl/fl) LysMcre-/-
HMGB1(fl/fl) LysMcre+
