# Supplementary material for: Tissue-specific inflammatory responses to systemic LPS in mice with LysM-lineage Hmgb1 deletion
Source: Brain Behav Immun Health. 2026 Oct 6;58:101382. doi: 10.1016/j.bbih.2026.101382 (PMC13677236; doi:10.1016/j.bbih.2026.101382)
Supplement: Supplementary file 2 [file mmc9.pdf]

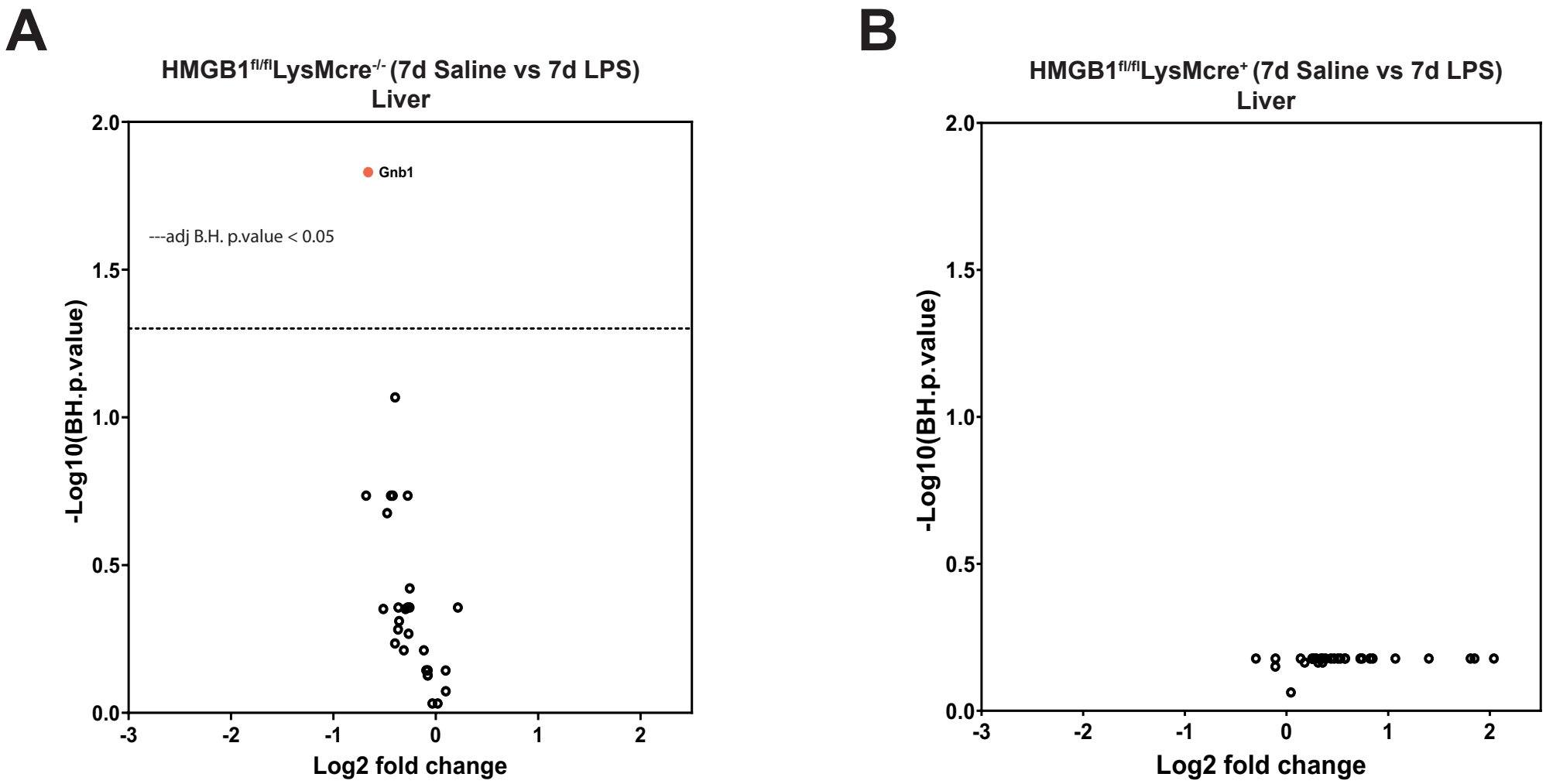
A
HMGB1fl/flLysMcre-/- (7d Saline vs 7d LPS)
Liver
Gnb1
---adj B.H. p.value < 0.05
-Log10(BH.p.value)
Log2 fold change
B
HMGB1fl/flLysMcre+ (7d Saline vs 7d LPS)
Liver
-Log10(BH.p.value)
Log2 fold change
