# Supplementary material for: Tissue-specific inflammatory responses to systemic LPS in mice with LysM-lineage Hmgb1 deletion
Source: Brain Behav Immun Health. 2026 Oct 6;58:101382. doi: 10.1016/j.bbih.2026.101382 (PMC13677236; doi:10.1016/j.bbih.2026.101382)
Supplement: Supplementary file 3 [file mmc1.docx]

**Supplementary Table S1. Reagents for all experiments**

| Primary Antibodies | Secondary Antibodies | Applications |
| --- | --- | --- |
| anti-CD45 rat  MCA1388, Bio-Rad (Hercules, CA) | goat anti-rat IgG (H+L) cross-adsorbed secondary antibody, Alexa Fluor™ 647 (#A-21247, RRID AB_141778)  Thermo Fisher Scientific (Waltham, MA) | CD45 immunoreactivity in the choroid plexus was assessed by immunofluorescence in Figure 6. |
| anti-cytokeratin mouse AB7753; Abcam, (Cambridge, MA) | donkey anti-mouse IgG (H+L) highly cross-adsorbed secondary antibody, Alexa Fluor™ 488 (#A-21202, RRID AB_141607)  Thermo Fisher Scientific (Waltham, MA) | Sections were co-stained with anti-cytokeratin antibody to delineate choroid plexus epithelial structures in Figure 6. |
| Chemical stains |  |  |
| DAPI (4′,6-diamidino-2-phenylindole 1 µg/mL; Sigma-Aldrich) | N/A | Nuclei were visualized using DAPI staining in Figure 6. |

**Supplementary Table S1A: Antibodies and chemical stains used in specific applications**.

**Supplementary Table S1B: Mouse primer sequences for qPCR.**

| Primer Name | Sequence |
| --- | --- |
| *mGzma* | Forward 5’-TGCTTATTCCTGAAGGAGGCTG-3’  Reverse 5’-CGCCAGCACAGATGGTATTTG-3’ |
| *mCd69* | Forward 5’-CTCCGTGGACCACTTGAGAG-3’  Reverse 5'-CTGGTGCCATGGTCCTTCTG-3’ |
| *mTNFα* | Forward 5'-TATGGCTCAGGGTCCAACTC-3'  Reverse 5'-CTCCCTTTGCAGAACTCAGG-3' |
| *mIl1b* | Forward 5′-TGAAGAAGAGCCCATCCTCTGTGA-3′ Reverse 5′-GGTCCGACAGCACGAGGCTT-3′ |
| *mCxcl10* | Forward 5′-CCACGTGTTGAGATCATTGCC-3’  Reverse 5′-TCACTCCAGTTAAGGAGCCC-3’ |
| *mGapdh* | Forward 5´-GAACATCATCCATGCATCCA-3´ Reverse 5´-CCAGTGAGCTTCCCGTTCA-3´ |

**Supplementary Table S1C: TaqMan probes.**

| Probe | Catalog Number |
| --- | --- |
| High mobility group box 1 (*Hmgb1*) | Mm04205650_gH |
| Glyceraldehyde 3-phosphate dehydrogenase (*Gapdh*) | Mm99999915_gI |

**Supplementary Table S1D: Additional reagents used in Methods**

| Reagent | Application |
| --- | --- |
| LPS (strain O111:B4; EMD Millipore Chemicals; Lot number D00153617; 5 mg/kg) | Used to induce systemic inflammation and experimental endotoxemia in mice |
| TRIzol  (15596018, Invitrogen) | RNA Isolation |
| Histopaque®‐1083 (Sigma‐Aldrich) | Bone marrow isolation |
| Maxima First Strand cDNA synthesis kit (K1641, Invitrogen) | RNA reverse transcription |
| PowerUp SYBR Green Master Mix (100029284, Thermo Fisher) | qPCR |
| TaqMan Fast Advanced Master Mix  (4444557, Thermo Fisher) | qPCR |
| Chloroform  (BP-1145-1, Fisher BioReagents) | RNA Isolation |
| Molecular grade ethanol  (BP2818-500, Fisher BioReagents) | RNA Isolation |
| Ambion Nuclease free water  (AM9932, Invitrogen) | RNA Isolation |
| Ambion DNA-free kit  (AM1906, Invitrogen) | RNA Isolation |
| Tissue Protein Extraction Reagent (T-PER) (78510, ThermoScientific) | Tissue homogenization during RNA isolation |
| Phosphatase inhibitor  (1862495, Thermo Scientific) | Added to tissue homogenizing solution with TPER at 1:100 dilution |
| Protease inhibitor  (78429, Thermo Scientific) | Added to tissue homogenizing solution with TPER at 1:100 dilution |
| Optimal cutting temperature compound (OCT) (4583, Sakura Finetec) | Used for fixed brain sectioning |
| Paraformaldehyde (PFA)  (1921, Electron Microscopy Sciences) | Used for brain fixation |
| Prolong gold  (P36930, Life Technologies, Eugene, OR) | Mounting media for immunofluorescent staining |
| Mouse TNF-α ELISA  (R&D Duoset Kit: DY410) | ELISA |
| Mouse IL-1β ELISA  (R&D Duoset Kit: DY401) | ELISA |
| Mouse HMGB1 ELISA  (CSB-E08225m, CUSABIO, Wuhan, China) | ELISA |

**Supplementary Table S1E: Genotyping Primers for *HMGB1^fl/fl^ LysM-Cre* Mice**

| Primer Name Sequence |  |
| --- | --- |
| olMR 3066 5’-CCC AGA AAT GCC AGA TTA CG-3’ |  |
| olMR 3067 5’-CTT GGG CTG CCA GAA TTT CTC-3’ |  |
| olMR 3068 5’-TTA CAG TCG GCC AGG CTG AC-3’ |  |
| HMGB1 flox Forward 5’-TGT CAT GCC ACC CTG AGC AGT T-3’ |  |
| HMGB1 flox Reverse 5’-TGT GCT CCT CCC GGC AAG TT-3’ |  |

**Supplementary Table S1F: Genotyping Reagents for *HMGB1^fl/fl^ LysM-Cre* Mice**

| Reagent | Application |
| --- | --- |
| EmeraldAmp Max PCR Master Mix  (RR320A, Takara) | Genotyping |
| Ambion Nuclease free water  (AM9932, Invitrogen) | Genotyping |
| UltraPure TAE Buffer, 10X  (15558042, Fisher Scientific) | Diluted to 1X TAE for genotyping |
| GeneRuler 100 bp Plus DNA Ladder,  ready-to-use  (SM0324, Thermo Scientific) | Genotyping |
| SYBR Safe DNA gel stain  (S33102, Invitrogen) | Genotyping |
| Agarose  (BP1356-500, Fisher BioReagents) | Used to make 1.5% agarose gel for genotyping |
| Qiagen DNeasy Blood & Tissue Kit  (69506, Qiagen) | Genotyping |

**Supplementary Table S1G: Expected PCR Products for *HMGB1^fl/fl^ LysM-Cre* Mice**

**Genotype Identification**

| Experimental genotype | Genotype | Expected Product | Interpretation |
| --- | --- | --- | --- |
| HMGB1 | HMGB1^fl/fl | 300 bp | Homozygous floxed |
| HMGB1 | HMGB1^fl/+ | 300 bp + 172 bp | Heterozygous floxed |
| HMGB1 | HMGB1^+/+ | 172 bp | Wild-type |
| LysM-Cre | LysM-Cre^+/+ | 700 bp | Homozygous Cre |
| LysM-Cre | LysM-Cre^+/− | 350 bp + 700 bp | Heterozygous Cre |
| LysM-Cre | LysM-Cre^− | 350 bp | Wild-type allele  (Cre-negative) |

**Note:** Experimental animals used in this study were HMGB1^fl/fl LysM-Cre^+/+ mice (homozygous for the LysM-Cre allele); genotype-matched controls were HMGB1^fl/fl LysM-Cre^− mice (Cre-negative)
