# Supplementary material for: Tissue-specific inflammatory responses to systemic LPS in mice with LysM-lineage Hmgb1 deletion
Source: Brain Behav Immun Health. 2026 Oct 6;58:101382. doi: 10.1016/j.bbih.2026.101382 (PMC13677236; doi:10.1016/j.bbih.2026.101382)
Supplement: Supplementary file 4 [file mmc7.docx]

**Supplementary Figure S1. Y-maze performance 7 days after systemic LPS exposure in mice with LysM-lineage Hmgb1 deletion.** Male *HMGB1^fl/fl^LysM-cre^+^* and *HMGB1^fl/fl^LysM-cre^-/-^* littermate control mice received a single intraperitoneal injection of LPS (5 mg/kg) or saline and were assessed in the Y-maze 7 days post-injection to evaluate later behavioral outcomes following systemic inflammation. (A) Total distance traveled (m) was used as a measure of locomotor activity. (B) Spontaneous alternation (%) was used as an index of spatial working memory. Data are presented as mean ± SEM (n = 9–11 mice per group). Statistical analysis was performed using two-way ANOVA with genotype and treatment as factors. No significant main effects of genotype or treatment and no genotype × treatment interaction were detected for either measure.

**Supplementary Figure S2. Liver transcriptional responses 7 days after LPS exposure in mice with LysM-lineage *Hmgb1* deletion.** Male *HMGB1^fl/fl^LysM-cre^+^* and *HMGB1^fl/fl^LysM-cre^-^* littermate control mice received a single intraperitoneal injection of LPS (5 mg/kg) or saline, and liver tissues were collected 7 days post-injection. Differential gene expression was assessed using the NanoString Mouse Inflammation Panel and analyzed with nCounter Advanced Analysis software. Volcano plots show differential gene expression following LPS relative to saline in *HMGB1^fl/fl^LysM-cre^-^* control mice (A) and *HMGB1^fl/fl^LysM-cre^+^* mice (B). Genes with a Benjamini–Hochberg (BH)-adjusted *P* value < 0.05 were considered significantly differentially expressed. In *HMGB1^fl/fl^LysM-cre^-^* control mice, *Gnb1* was significantly downregulated following LPS relative to saline. No genes met the BH-adjusted *P* < 0.05 significance criterion in *HMGB1^fl/fl^LysM-cre^+^* mice. Log₂ fold change indicates the direction and magnitude of gene-expression changes and was not applied as an additional significance threshold. n = 3 biological samples per genotype × treatment group.
